# CO_2_‐to‐CO Electrolysis in Pure Water at Ampere‐Level Current Density and 1000 h Stability via a Rapid‐Transport Fixed‐Charge Interface

**DOI:** 10.1002/advs.75379

**Published:** 2026-04-22

**Authors:** Qiqi Wan, Gang Zhu, Wenxing Jiang, Yingying Liu, Jie Gui, Chi Xu, Ji Chen, Xiaodong Zhuang, Changchun Ke

**Affiliations:** ^1^ Institute of Fuel Cells School of Mechanical Engineering Shanghai Jiao Tong University Shanghai P. R. China; ^2^ Wuhan Institute of Marine Electric Propulsion Wuhan P. R. China; ^3^ Shanghai Phoenix Technology Co., Ltd Shanghai P. R. China; ^4^ School of Chemistry and Chemical Engineering Shanghai Jiao Tong University Shanghai P. R. China

**Keywords:** CO_2_ electrolysis, fixed‐charge interface, interfacial engineering, mass transport, pure water

## Abstract

Electrochemical reduction of CO_2_ to CO offers a sustainable pathway to syngas for synthetic fuels and chemicals. Conducting CO_2_ electrolysis in pure water simplifies system design and avoids salt precipitation, yet its performance is constrained by sluggish reaction kinetics and limited selectivity. To address these challenges, we constructed a rapid‐transport fixed‐charge interface (RTFC‐I) via electrochemical reconstruction of a polymer‐modified Ag electrode, yielding a nanostructured surface coated with a quaternary ammonium polymer layer. This design creates rapid mass transport channels and a positively charged microenvironment, which stabilizes critical reaction intermediates while restricting proton transport. The optimized electrode achieves a CO Faradaic efficiency (FECO) of ∼99% at 500 mA cm^−2^ (25°C), and maintains 76.4% FECO at 1.0 A cm^−2^ (60°C). It also exhibits stable continuous operation for 1000 h, along with excellent scalability, as validated in an integrated three‐cell stack featuring a total active area of 960 cm^2^. In situ Fourier‐transform infrared (FTIR) spectroscopy, differential electrochemical mass spectrometry (DEMS), and density functional theory (DFT) calculations verify that the RTFC‐I reduces the energy barrier for *COOH formation and enhances CO_2_ reduction activity. This work validates fixed‐charge nanostructured interfaces as a robust strategy for alkaline‐free CO_2_ electrolysis in pure water.

## Graphical abstract

CO_2_ reduction in pure water is enabled by a rapid‐transport fixed‐charge interface that couples nanochannels with a cationic microenvironment. This design eliminates the mass transport plateau, stabilizes key reaction intermediates, and suppresses the hydrogen evolution reaction, ultimately achieving ampere‐level current densities and stable operation for over 1000 h.

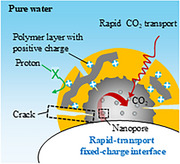

## 1 Introduction

Electrochemical reduction of CO_2_ (CO_2_RR) to CO provides a sustainable route to synthetic fuels and chemicals [1, 2], as CO is a key component of syngas used in industrial processes such as Fischer‐Tropsch synthesis [3, 4]. To achieve high CO_2_RR current density and CO Faradaic efficiency (FECO), state‐of‐the‐art CO_2_‐to‐CO electrolyzers predominantly use alkaline electrolytes [5, 6, 7, 8], such as KOH or KHCO_3_ solutions. However, the use of these electrolytes introduces salt precipitation in gas flow channels and the porous transport layer of the cathode, typically limiting continuous operation to several tens or hundreds of hours, thereby significantly constraining the durability and industrial scalability of CO_2_RR systems [9, 10, 11].

**SCHEME 1:**
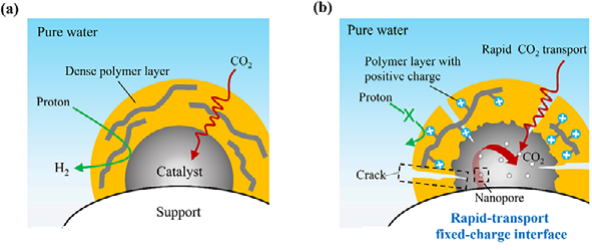
Schematic illustration of catalytic interface designs for CO_2_RR in pure water. (a) A conventional catalytic surface covered by a dense polymer layer. (b) The rapid‐transport fixed‐charge interface (RTFC‐I) featuring nanochannels, which maintains a cationic microenvironment in pure water (alkaline‐free) while enabling efficient CO_2_ transport.

Operating CO_2_RR in pure water emerges as an appealing alternative, as it circumvents the undesired formation and precipitation of carbonates. For instance, a zero‐gap electrolyzer integrated with amino‐functionalized Ag catalysts, using pure water as the anolyte, achieved a FECO of 95.5% at 250 mA cm^−2^ (50°C) and sustained FECO above 93% over 180 h [12]. In another example, an Ag cathode modified with immobilized poly(diallyldimethylammonium chloride)‐graphene oxide achieved 100 mA cm^−2^ with 78% FECO and 200 mA cm^−2^ with 55% FECO in pure water [13]. Despite these advances, most reported CO_2_‐to‐CO systems operating in pure water still exhibit lower current densities and selectivity compared to those using alkaline electrolytes [7, 14, 15]. A primary limitation stems from conventional electrode designs that rely on dense polymer layers at the catalyst surface. Although such layers establish a favorable local electrostatic environment, they also form compact barriers that restrict CO_2_ and ion access to active sites, resulting in pronounced mass transport limitations at high current densities (Scheme 1a).

In this work, a fixed‐charge nanostructured interface is designed to simultaneously overcome both the kinetic and mass transport limitations of CO_2_RR in pure water. A dense fixed‐charge poly(diallyldimethylammonium) (PDDA) layer was electrochemically reconfigured into a rapid‐transport fixed‐charge interface (RTFC‐I) with a nanoporous transport network, while preserving its favorable positively charged microenvironment (Scheme 1b). This resultant nanostructure affords short diffusion pathways, thereby eliminating the mass transport plateau, while the immobilized positive charges stabilize critical reaction intermediates and effectively suppress the competing hydrogen evolution reaction (HER). Consequently, this work achieves high‐performance CO_2_‐to‐CO electrolysis in pure water, demonstrating ampere‐level current density, enhanced long‐term operational durability, and stack‐level scalability.

## 2 Results and Discussion

### 2.1 Performance of Rapid‐Transport Fixed‐Charge Interface (RTFC‐I) in Pure Water

As depicted in Scheme 1b, the Ag‐based RTFC‐I integrates a nanostructured catalyst surface with a quaternary ammonium polymer (PDDA, 6.19 mmol g^−1^) [16], which provides a fixed positively charged interface. RTFC‐I is generated from DFC‐I through a brief electrochemical oxidation‐reduction reconstruction process. Specifically, the initial single‐crystalline Ag nanoparticles are first partially oxidized to Ag_2_O under anodic potential, which is accompanied by volume expansion, and are then rapidly reduced back to metallic Ag under cathodic potential. This expansion‐contraction process induces local stress, leading to the formation of void and crack features within the catalyst layer, thereby creating a rapid‐transport catalytic interface. RTFC‐I is designed to maintain the electrostatic benefits of the positively charged environment while alleviating the mass transport bottleneck that typically occurs at high current densities. The detailed electrochemical reconstruction protocol is described in the Experimental Section.

**FIGURE 1:**
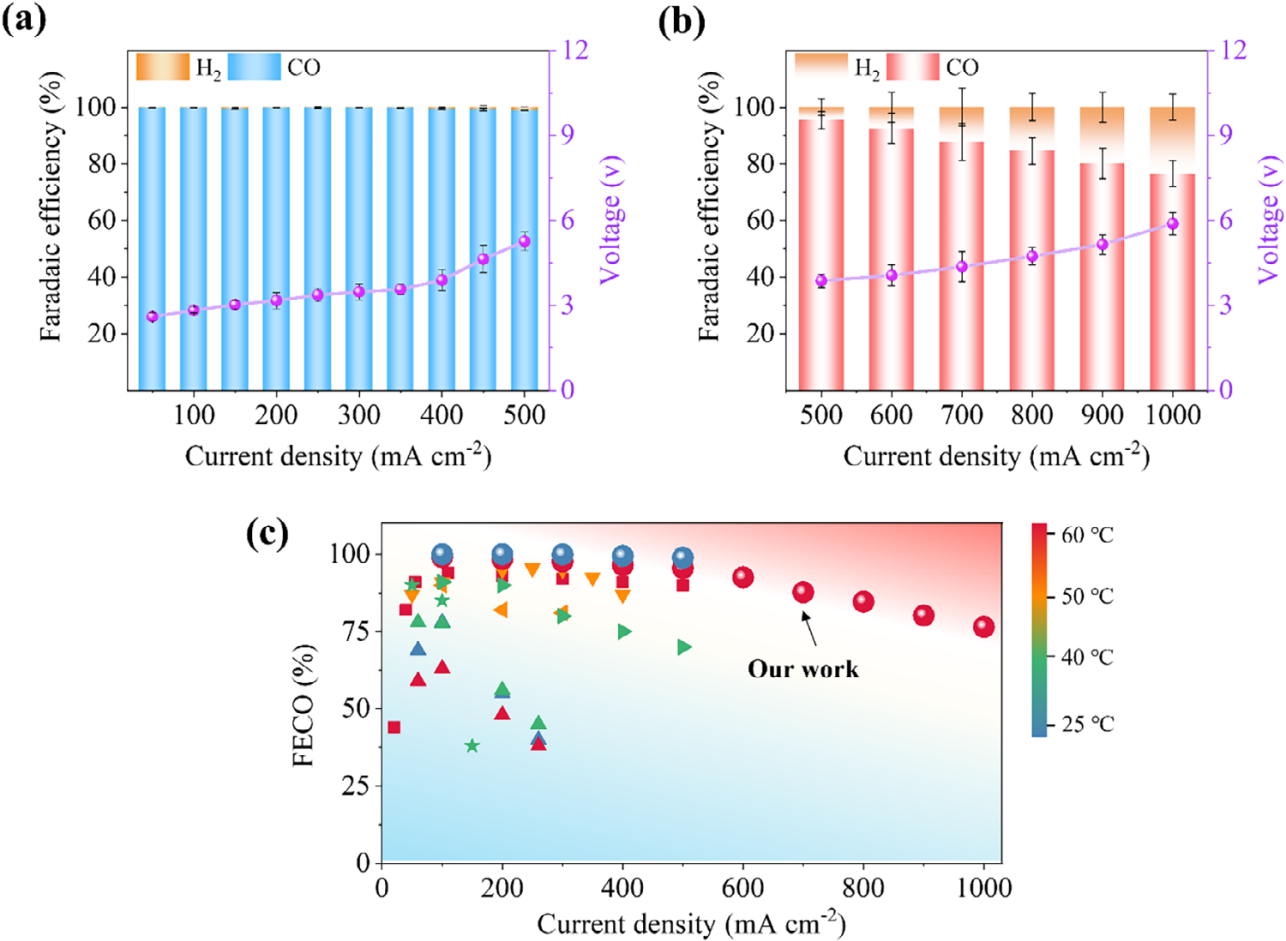
Electrochemical performance of the RTFC‐I for CO_2_RR in pure water. FECO and cell voltage as a function of current density at a) 25°C and b) 60°C, reaching 1.0 A cm^−2^ with high FECO. c) Benchmark comparison of FECO versus current density between this work (solid spheres) and previously reported state‐of‐the‐art studies for CO_2_RR operating in pure water [12, 13, 14, 15, 16, 17, 18, 19, 20]. Different symbols represent individual studies, and colors indicate operating temperature. Detailed data and references are listed in Table S1.

A zero‐gap electrolyzer using pure water as the anolyte was employed to evaluate RTFC‐I performance in CO_2_RR. The product distribution across various current densities reveals that CO and H_2_ are the sole detectable products. As shown in Figure 1a, when the applied current density increases from 50 to 500 mA cm^−2^ at 25°C, FECO remains extremely high. Specifically, the FECO exceeds 99% up to 400 mA cm^−2^ and is maintained at 98.9% even at 500 mA cm^−2^, while the cell voltage rises from 2.60 to 5.36 V. This corresponds to a maximum CO partial current density of 494.5 mA cm^−2^. Thus, RTFC‐I achieves high current densities with almost exclusive CO formation, whereas conventional electrodes typically suffer from rapid loss of FECO (Figure S1a‐c).

To probe the upper performance limit, the operating temperature was raised to 60°C, a common condition for CO_2_RR in pure water. Under these conditions, RTFC‐I maintains high FECO over a wide current density range and achieves an ampere‐level current density (Figure 1b; Figure S2). Specifically, a maximum CO partial current density of 764.5 mA  cm^−2^ is recorded at a total current density of 1.0  A  cm^−2^ with 76.4 %  FECO, which sets a new benchmark for CO_2_‐to‐CO conversion in pure water.

As illustrated in Figure 1c (with details provided in Table S1), the performance achieved in our work represents a significant advance for CO_2_RR in pure water, particularly at high current densities. By achieving ampere‐level current densities and near‐exclusive CO formation, this work effectively bridges the performance gap between systems operating in pure water and those employing supporting electrolytes. Notably, the performance at 25°C is already on par with the best results previously reported in the literature, which often required elevated temperatures to enhance reaction kinetics.

### 2.2 Characterization of RTFC‐I

To investigate why achieving high current densities (> 300 mA cm^−2^) is challenging in pure water systems, we compared the polarization behavior of conventional and rapid‐transport interfaces. A regular catalytic interface covered by a dense polymer layer with fixed positive charge was first prepared as a baseline, denoted as DFC‐I (dense fixed‐charge interface). A brief electrochemical reconstruction step then converted this dense layer into a rapid‐transport fixed‐charge interface, denoted as RTFC‐I.

**FIGURE 2:**
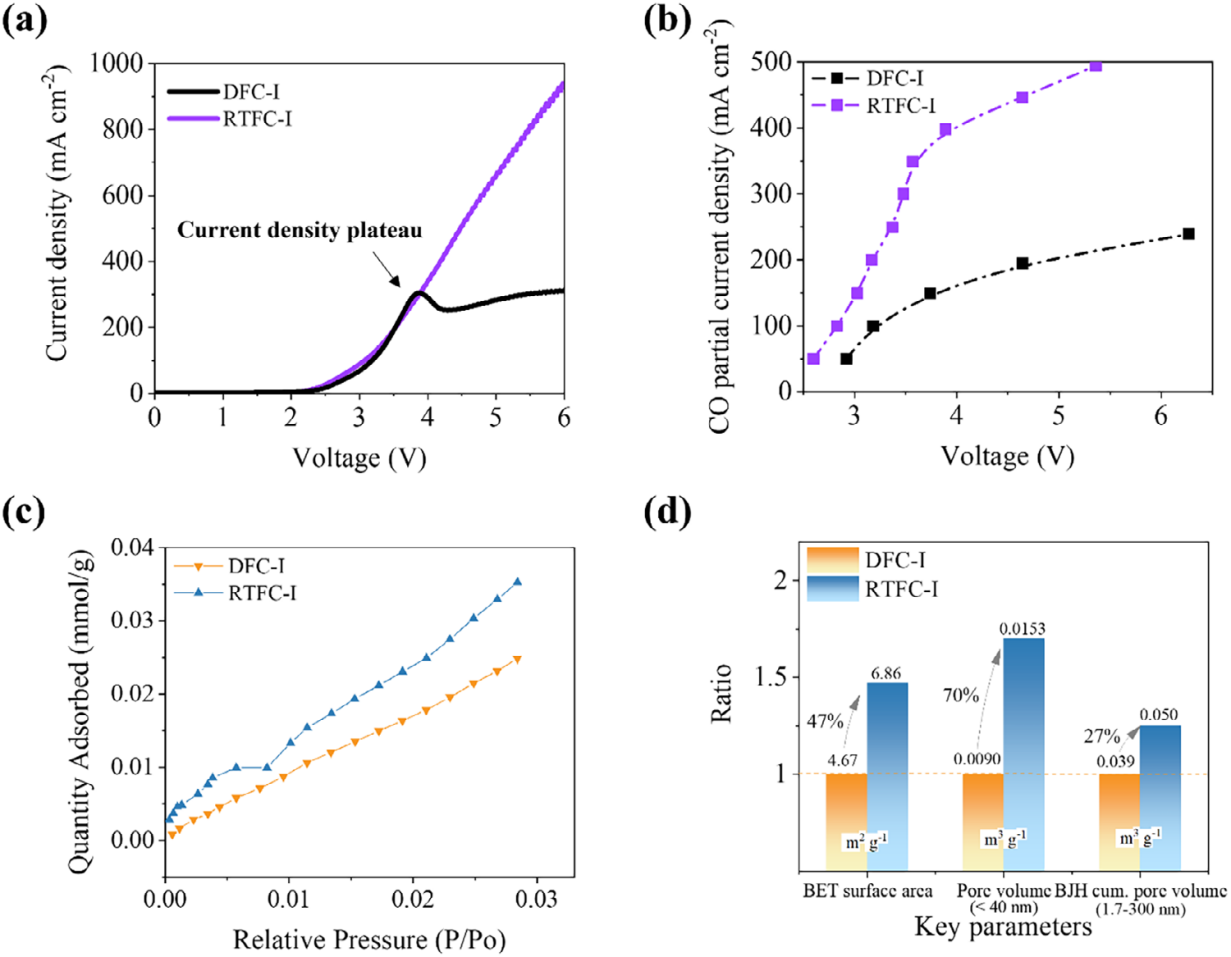
Electrochemical and physical characterization for elucidating enhanced transport in RTFC‐I. (a) Linear sweep voltammetry (LSV) curves. (b) CO partial current density as a function of cell voltage, in which symbols represent the experimentally measured data points, and the dash‐dotted lines are fitted curves. The electrochemical data were acquired at 25°C. (c) CO_2_ adsorption isotherms at 25°C and (d) comparison of BET surface area and pore volume between a dense interface (DFC‐I) and a nanostructured interface (RTFC‐I).

The DFC‐I operating in pure water exhibits a broad current plateau in its linear sweep voltammetry (LSV) curves (Figure 2a), indicating that mass transport becomes a limiting factor even below 500 mA cm^−2^. In contrast, the plateau disappears for RTFC‐I, with the current density increasing smoothly with cell voltage. Concomitantly, the CO partial current density is significantly enhanced (Figure 2b). Complementary measurements reveal an increase in double‐layer capacitance (Figure S3), indicating a larger electrochemically active surface area, and a decrease in charge transfer and mass transport resistances as evidenced by electrochemical impedance spectroscopy (Figure S4). These observations are consistent with the alleviation of the mass transport bottleneck.

To test the generality of this interface design, PDDA was first replaced by PiperION to prepare a PiperION‐based electrode. Similar to the PDDA‐based system, the current plateau likewise disappears after reconstruction (Figure S5), and the reconstructed electrode sustains a current density of 200 mA cm^−2^, compared with 150 mA cm^−2^ before reconstruction (Figure S6). In addition, a PDDA‐based Cu electrode was also prepared. After reconstruction, the formation of CO and C_2_H_4_ increases, while HER is suppressed (Figure S7), collectively suggesting that the strategy of creating rapid mass transport channels is not limited to the current system.

Gas adsorption measurements corroborate that RTFC‐I provides a more open transport network for CO_2_. CO_2_ adsorption isotherms at 25°C show a consistently higher uptake for RTFC‐I than DFC‐I over the entire pressure range (Figure 2c), indicating more accessible sorption sites and pore volume. BET analysis derived from N_2_ adsorption‐desorption isotherms reveals significant increases in specific surface area, total pore volume (for pores <40 nm), and BJH cumulative pore volume (1.7–300 nm) after reconstruction (Figure 2d), suggesting the formation of nanoscale voids. These structural features provide a physical basis for the disappearance of the current plateau in LSV and the enhanced CO_2_RR performance of RTFC‐I at high current densities in pure water.

Despite the similar improvement in transport, product selectivity responds differently under distinct charge environments. RTFC‐I maintains ∼99% FECO from 50 to 500 mA cm^−2^ (Figure 1a). In contrast, for the PiperION‐based electrode with nanochannels (2.3–3.5 mmol g^−1^, lower fixed‐charge density) [21], the increased current after reconstruction is largely diverted to HER as FECO drops at higher current densities (Figure S8). These results show that constructing a rapid transport interface is a general strategy to remove mass transport limitations, but only when coupled with a strongly fixed‐charge microenvironment (such as PDDA) can high CO selectivity and high activity be achieved simultaneously.

**FIGURE 3:**
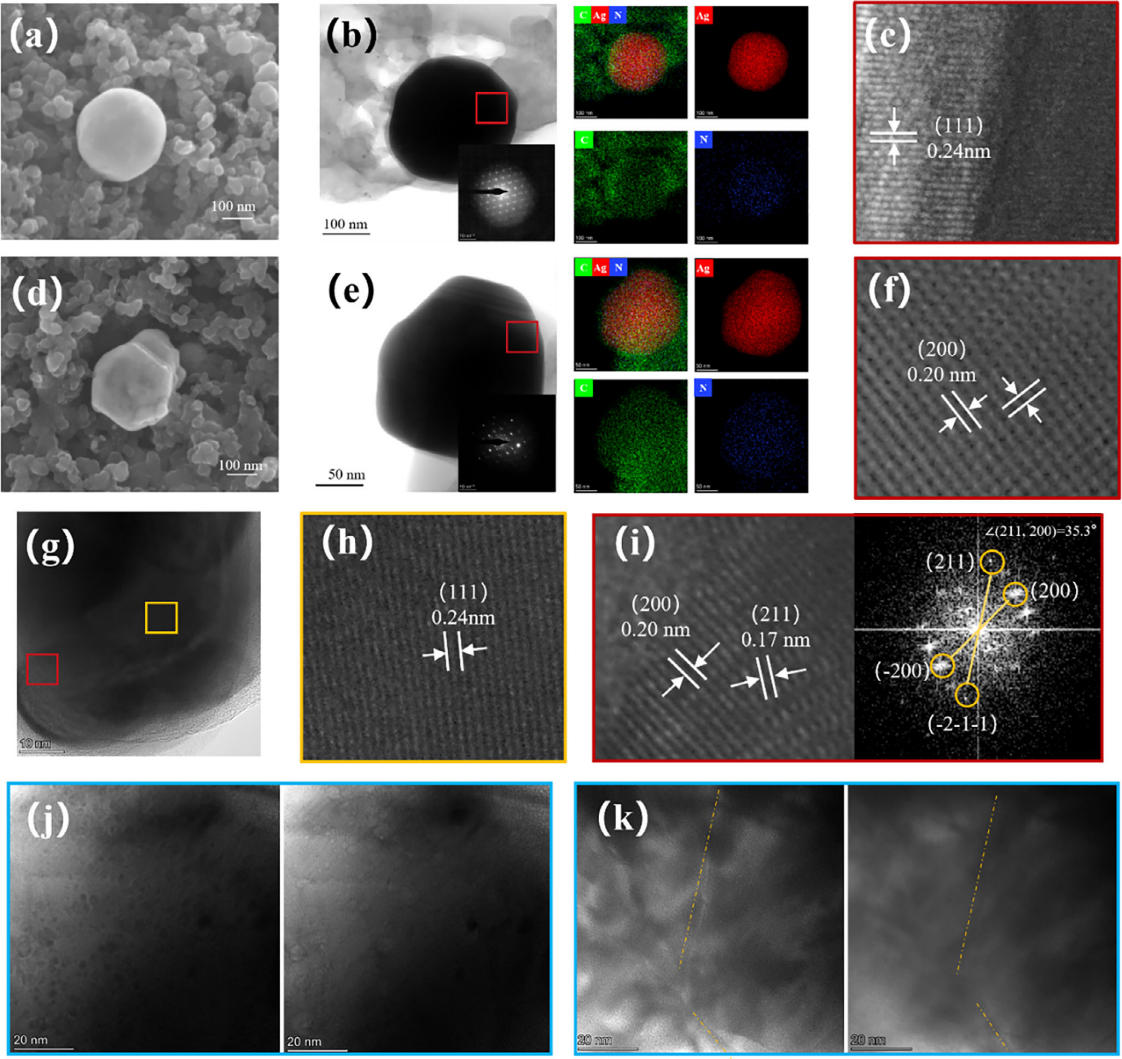
Morphological and structural evolution of the catalytic interface. SEM images of (a) DFC‐I and (d) RTFC‐I. TEM images of representative Ag particles in (b) DFC‐I and (e) RTFC‐I; insets show the corresponding SAED patterns; the right‐hand panels display EDS maps for Ag, C, and N. HRTEM images revealing the lattice fringes of (c) DFC‐I and (f) RTFC‐I. Further analysis of a particle in RTFC‐I, where (h) and (i) are magnified views of the regions marked in (g). HRTEM images with Fresnel contrast inversion; the reversal of contrast from over‐focus (left) to under‐focus (right) confirms the presence of (j) true cavities and (k) surface cracks.

Having confirmed that RTFC‐I eliminates the mass transport plateau and enhances CO_2_ uptake, the underlying structural changes were examined. Scanning electron microscopy (SEM) images of DFC‐I show spherical Ag nanoparticles homogeneously mixed with carbon black (Figure 3a; Figure S9). In contrast, the particles in the RTFC‐I develop a more distinct and faceted morphology (Figure 3d; Figure S10).

High‐resolution transmission electron microscopy (HRTEM) further highlights the evolution. For DFC‐I, the selected‐area electron diffraction (SAED) pattern of a representative Ag particle exhibits clear spots consistent with a single face‐centered cubic (fcc) crystal (Figure 3b; Figure S11). The corresponding HRTEM image shows lattice fringes with a spacing of ≈0.24 nm, characteristic of Ag(111) planes (Figure 3c) [22]. For RTFC‐I, the SAED pattern changes from discrete spots to concentric rings, indicating that the initial single‐crystalline Ag nanoparticles become polycrystalline after reconstruction (Figure 3e; Figure S12). HRTEM images of the reconstructed interface reveal additional sets of fringes with spacings of ≈0.20 and ≈0.17 nm that can be attributed to Ag(200) and Ag(211) planes [23], respectively (Figure 3f‐i). The measured angle between the (200) and (211) fringes agrees with the theoretical value for fcc Ag, indicating that the reconstruction generates polycrystalline particles, and (211) planes can be identified in local HRTEM projections, which is consistent with the formation of local stepped structures.

The origin of the enhanced transport in RTFC‐I is attributed to the expansion‐contraction sequence during a brief anodic‐cathodic treatment. A short anodic polarization partially oxidizes Ag to Ag_2_O, inducing local volume expansion [24, 25]; the subsequent cathodic step rapidly reduces the oxide and contracts the structure within seconds (Figure S13). This sequence generates nanoscale voids and surface cracks within the catalyst layer. These features can be directly visualized via Fresnel‐contrast imaging, a defocus‐based TEM method in which images are intentionally recorded slightly over‐focus and under‐focus [26, 27]. Under these conditions, changes in projected thickness or potential at the edge of a void or crack generate bright/dark fringe contrast, and the inversion of this contrast upon changing the sign of defocus is characteristic of true open features. In Figure 3j,k, the Fresnel‐contrast imaging reveals numerous sub‐5 nm cavities and cracks, whose contrast inverts upon switching from over‐focus to under‐focus conditions, a characteristic of true open features rather than imaging artefacts (see Figure S14 for additional examples). Combined with the increased surface area and pore volume from gas adsorption analysis (Figure 2d; Figure S15 and Table S2), these observations demonstrate that the RTFC‐I has been transformed into a porous morphology with stepped local structures. This nanostructure provides both additional active sites and shortened diffusion pathways, offering a structural rationale for the elimination of the current plateau and the achievement of high CO partial current density in pure water.

Furthermore, X‐ray photoelectron spectroscopy (XPS, Figure S16) and X‐ray diffraction (XRD, Figure S17) confirm that the bulk chemical state of Ag and the PDDA layer remain essentially unchanged. This indicates that the performance enhancement originates primarily from the morphological and interfacial design, rather than a change in phase composition.

### 2.3 Mechanistic Origin of Enhanced CO_2_‐to‐CO Conversion

**FIGURE 4:**
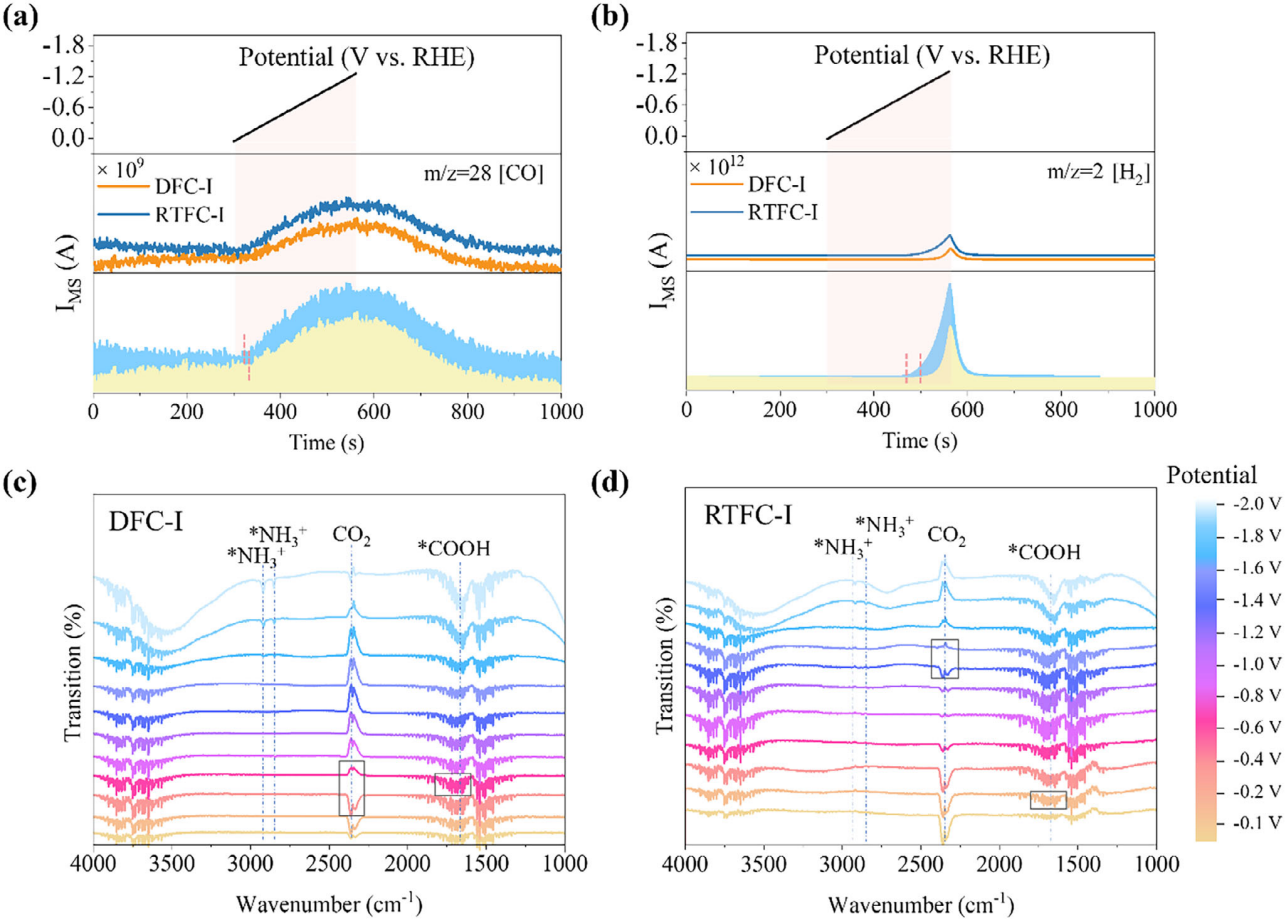
In situ spectroscopic analysis. (a,b) Differential electrochemical mass spectrometry (DEMS) results, tracking the signals of CO and H_2_. For each subplot, the top panel shows the applied potential, the middle panel displays the corresponding mass spectrometric ion current for the products, and the bottom panel provides a magnified view of the low‐signal region. In situ Fourier‐transform infrared (FTIR) spectra recorded at various applied potentials for PDDA‐based (c) DFC‐I and (d) RTFC‐I. The highlighted regions emphasize key changes upon reconstruction: the delayed inversion of the CO_2_ band and the significantly enhanced *COOH signal intensity.

To clarify how RTFC‐I modulates reaction pathways, differential electrochemical mass spectrometry (DEMS) was used to track the signals of CO (m/z = 28) and H_2_ (m/z = 2) as a function of potential for PDDA‐based and PiperION‐based electrodes in dense and reconstructed states (Figure 4a,b; Figure S18a,b). The specific DEMS configuration is shown in Figure S19. Upon applying a series of millivolt‐scale potential steps, the reconstructed interfaces for both electrode types show comparable onset potentials for CO_2_‐to‐CO conversion, and the onset potential of HER shifts positively after reconstruction, which is consistent with the polarization curves.

A clear distinction emerges, however, in the product selectivity. For the PiperION‐based electrode, reconstruction leads to a substantially larger H_2_ signal, demonstrating that much of the additional current is diverted toward the competing HER pathway (Figure S18b). In contrast, for the PDDA‐based RTFC‐I, the increase in the H_2_ signal is modest, while the CO signal exhibits a more pronounced enhancement over the same potential range (Figure 4a). This is consistent with the high FECO maintained from 50 to 1000 mA cm^−2^. These DEMS results collectively indicate that the rapid‐transport reconstruction serves as a general strategy to increase apparent activity. However, a strong, fixed‐charge microenvironment, as provided by PDDA, is essential to direct the enhanced activity toward CO_2_RR rather than the HER.

To monitor surface species during CO_2_RR, in situ Fourier‐transform infrared spectroscopy (FTIR) was performed on DFC‐I and RTFC‐I (Figure 4c,d; the configuration is shown in Figure S20). Two bands at ≈ 2915 and ≈ 2843 cm^−1^, characteristic of C‐H stretching modes [12], confirm the presence of the quaternary ammonium polymer and thus a fixed‐charge environment. In Figure 4c,d, the background CO_2_ band at ≈2343 cm^−1^ [28] is retained to compare the initial CO_2_ adsorption capability of DFC‐I and RTFC‐I under the same feeding condition. The stronger CO_2_ signal of RTFC‐I indicates stronger initial CO_2_ adsorption at the reconstructed interface, consistent with the independent CO_2_ adsorption result in Figure 2c. With increasing cathodic potential, this band gradually inverts from negative to positive, and the inversion potential shifts from −0.6 V on DFC‐I to −1.4 V on RTFC‐I, indicating that RTFC‐I delays the onset of mass transport limitation. Background‐subtracted spectra are additionally provided in Figure S21. In these spectra, obvious CO_2_ consumption appears at about −0.6 V for DFC‐I but already at about −0.2 V for RTFC‐I, indicating earlier activation of CO_2_RR on RTFC‐I. The band around ≈1647 cm^−1^, which may contain contributions from *COOH as well as water bending modes and carbonate species [29], shows the same trend, appearing earlier and more strongly on RTFC‐I. This correlation supports its assignment to *COOH. Therefore, the earlier appearance of *COOH indicates accelerated interfacial CO_2_RR kinetics, whereas the delayed inversion of the CO_2_ band indicates postponed mass transport limitation.

Furthermore, similar but much weaker features for both CO_2_ and the *COOH intermediate are observed on the PiperION‐based electrodes (Figure S18c,d). This indicates that achieving simultaneous enhancement in kinetics and mass transport requires the combination of a strong fixed‐charge microenvironment with the engineered nanochannels.

**FIGURE 5:**
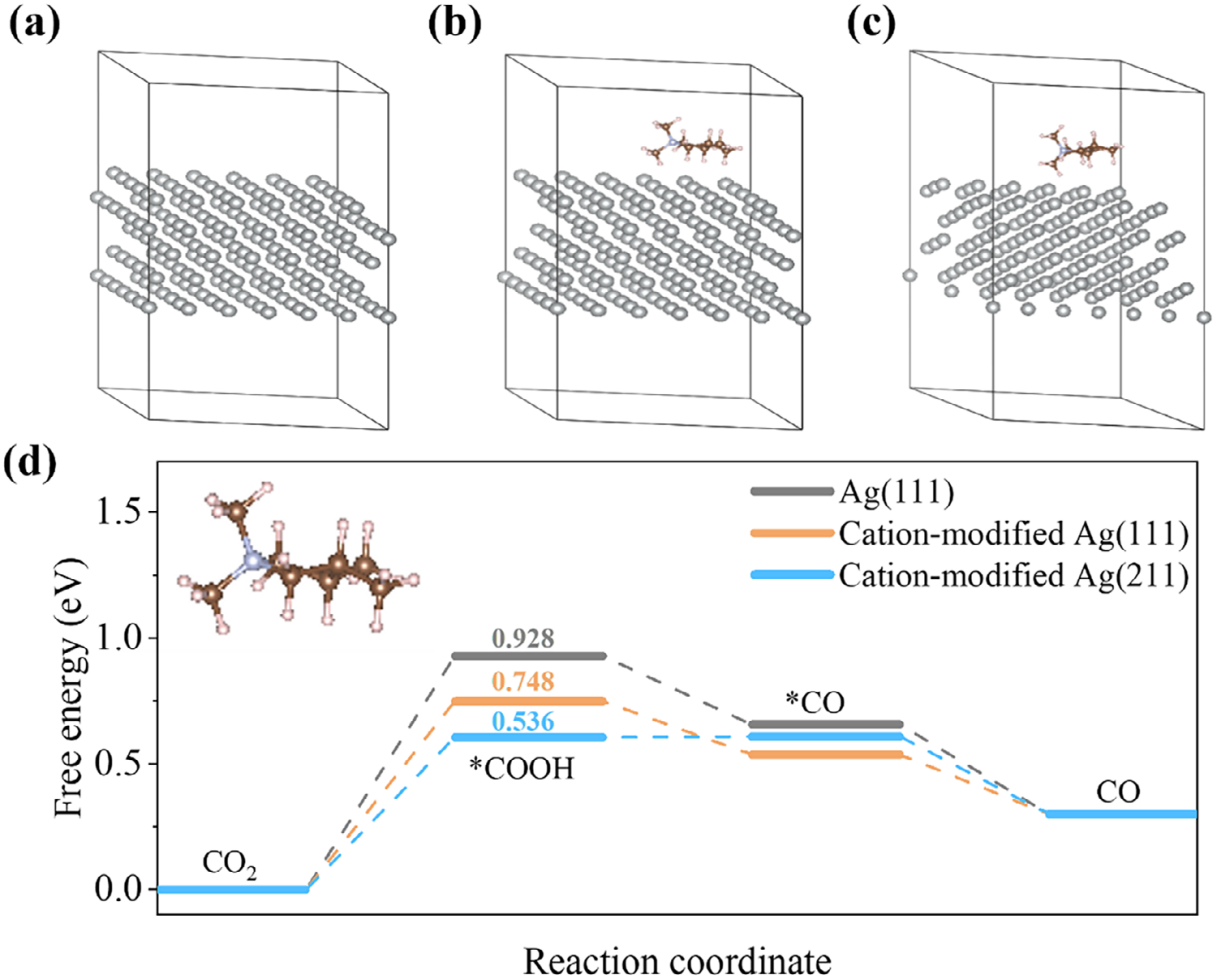
DFT modeling of the catalytic interfaces. Atomistic slab models used in the calculation: (a) bare Ag(111); (b) Ag(111) with a proximal quaternary ammonium cation (representing the DFC‐I); (c) Ag(211) with the same cation (representing the RTFC‐I). (d) Corresponding Gibbs free energy diagrams for the CO_2_‐to‐CO pathway.

To probe the mechanism of the enhanced CO_2_RR activity of RTFC‐I at the atomic scale, density functional theory (DFT) calculations were performed to evaluate the energetics of key intermediates. Three model surfaces are considered to represent the facet structure and fixed charge: bare Ag(111) representing a conventional metallic surface, cation‐modified Ag(111) representing the dense fixed‐charge interface (DFC‐I), and cation‐modified Ag(211) serving as a representative stepped surface for the reconstructed rapid‐transport fixed‐charge interface (RTFC‐I) (Figure 5a‐c; Figures S22‐S24). For all models, the CO_2_‐to‐CO pathway is described as two coupled proton/electron transfers: CO_2_ + H^+^ + e^−^ → *COOH, followed by *COOH + H^+^ + e^−^ → *CO + H_2_O, where * denotes an adsorbed species.

From the computed free energy diagrams, the formation of *COOH is the rate‐determining step for all models, consistent with the literature [30, 31, 32]. As depicted in Figure 5d, the cationic field on Ag(111) moderately stabilizes the *COOH intermediate relative to bare Ag(111), lowering the free energy barrier from 0.928 to 0.748 eV. When the same fixed‐charge environment is combined with stepped local structures, the free energy for *COOH formation is further reduced from 0.748 to 0.536 eV, representing the most favorable thermodynamics among the three models. This result provides an atomistic explanation for the experimental observations, as evidenced by the earlier and more intense appearance of the *COOH band in FTIR for the RTFC‐I.

In conjunction with the transport advantages confirmed by the nanostructured morphology, the RTFC‐I thus simultaneously optimizes the local atomic structure and interfacial electrostatics to lower the kinetic barrier for CO_2_RR in pure water.

### 2.4 Long‐Term Durability and Scalability

**FIGURE 6:**
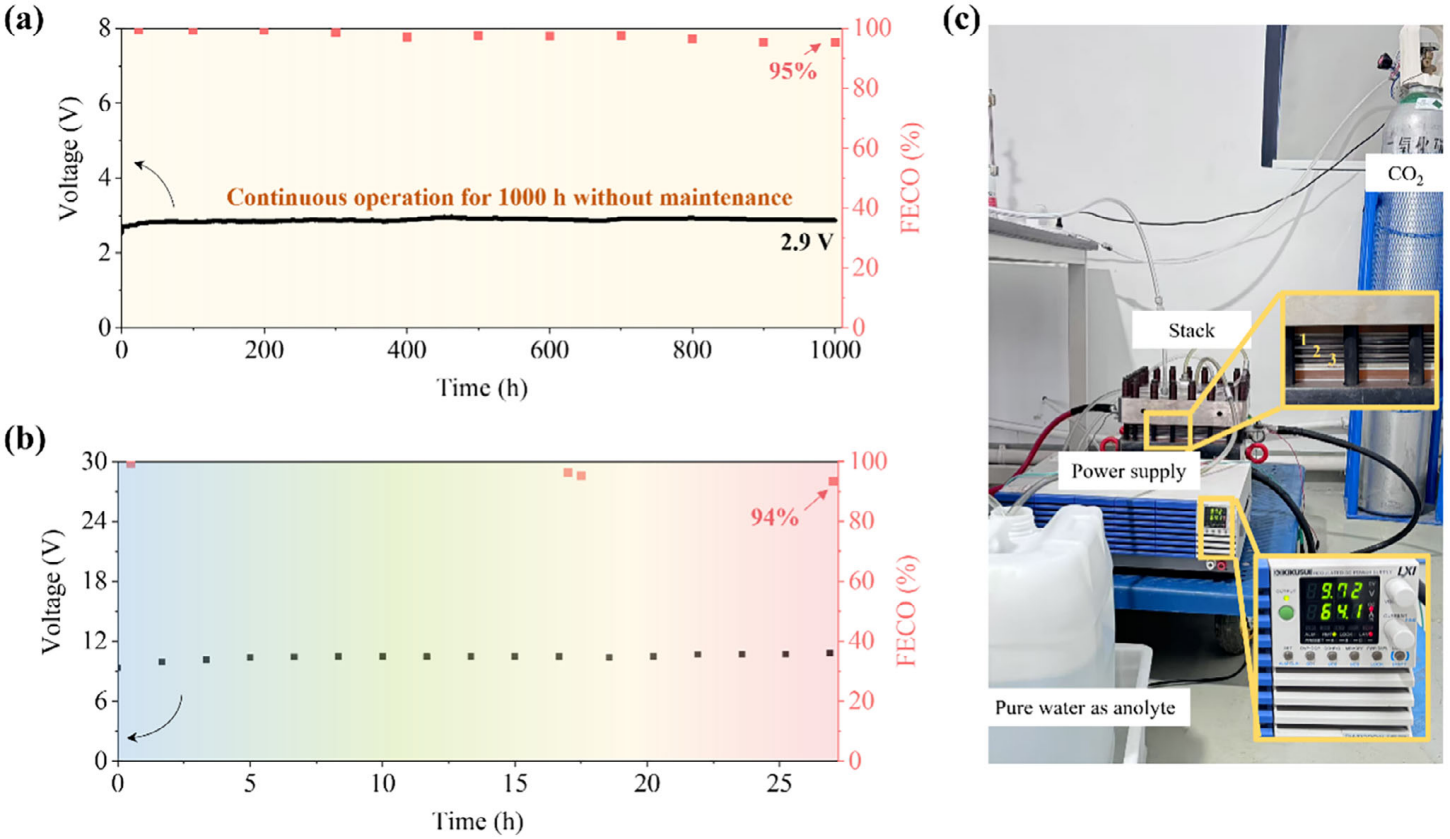
Long‐term durability and stack‐level scalability. a) Long‐term durability test in an 80 cm^2^ electrolyzer at 100 mA  cm^−2^ (25°C), demonstrating stable cell voltage and FECO above 95% over 1000 h. b) Performance of a three‐cell stack (320 cm^2^ per cell; total active area = 960 cm^2^) operated at 200 mA cm^−2^ and 25°C. c) Photograph of the operating three‐cell stack during continuous CO_2_RR.

The long‐term durability and scalability of RTFC‐I were evaluated. First, an electrolyzer with an active area of 80 cm^2^ was operated at an industrially relevant 100 mA cm^−2^ at 25°C (Figure 6a; Figure S25), corresponding to a total current of 8 A. Over 1000 h of continuous operation, the cell voltage remains ∼2.9 V with >95% FECO. Furthermore, the electrolyzer was operated at 16 A (200 mA cm^−2^) for 150 h, during which no significant performance degradation was observed (Figure S26). Post‐stability SEM, XPS, and XRD characterizations were further performed after the durability test (Figures S27‐S29). These results, together with the durability data, indicate that RTFC‐I remains structurally and electrochemically stable during prolonged high current operation.

To further assess scalability, a three‐cell stack (series connection; 320 cm^2^ per cell; Figure 6b) was assembled and tested. The stack exhibits comparable performance to the laboratory‐scale 4 cm^2^ electrolyzer (Figure S30). During durability testing, it shows a total current of 64 A (200 mA cm^−2^) with FECO exceeding 94% over 27 h (Figure 6c, details in Figures S31‐S33 and Video S1). The test was terminated due to depletion of the CO_2_ gas supply, not performance degradation. This successful scale‐up to a multi‐cell stack configuration confirms that coupling geometric reconstruction with a fixed‐charge interfacial microenvironment has achieved durable, high‐rate CO_2_RR in pure water and is directly translatable from the laboratory to industrially relevant stack‐level systems.

## 3 Conclusion

Here we report a practical strategy for high‐performance CO_2_ reduction to CO in pure water via a rapid‐transport fixed‐charge interface (RTFC‐I). A simple electrochemical reconstruction step converts a dense polymer layer into a nanoporous fixed‐charge interface, which simultaneously creates local stepped Ag structures and efficient mass transport nanochannels, while preserving a positively charged microenvironment that stabilizes key intermediates while restricting proton access. This synergistic design effectively mitigates mass transport limitations and reduces the kinetic barrier for CO_2_RR.

The resulting electrolyzer delivers a CO Faradaic efficiency of ∼99% at 500 mA cm^−2^ (25°C) and maintains 76.4% FECO at 1.0 A cm^−2^ (60°C). Stable operation for over 1000 h with >95% FECO is achieved in an 80 cm^2^ zero‐gap electrolyzer, and scalability is demonstrated in a three‐cell stack (total active area 960 cm^2^) with comparable performance.

In summary, this work demonstrates that integrating engineered nanochannels with a fixed‐charge microenvironment can transform conventional electrodes into highly active and durable interfaces for CO_2_RR. The RTFC‐I design offers a generalizable principle for achieving durable, high‐rate, and alkaline‐free CO_2_‐to‐CO conversion in pure water, providing a feasible path toward industrially relevant CO_2_ electrolysis.

## Conflicts of Interest

The authors declare no conflicts of interest.

## Supporting information




**Supporting File**: advs75379‐sup‐0001‐SuppMat.docx.

**Supporting Movie 1**: advs75379‐sup‐0002‐MovieS1.mp4.

## Data Availability

The data that supports the findings of this study are available in the supplementary material of this article.
