# Supplementary material for: CO2‐to‐CO Electrolysis in Pure Water at Ampere‐Level Current Density and 1000 h Stability via a Rapid‐Transport Fixed‐Charge Interface
Source: Adv Sci (Weinh). 2026 Apr 22;13(40):e75379. doi: 10.1002/advs.75379 (PMC13335526; doi:10.1002/advs.75379)
Supplement: Supplementary file 1 — Supporting File: advs75379‐sup‐0001‐SuppMat.docx. [file ADVS-13-e75379-s002.docx]

Supporting Information

**CO_2_-to-CO Electrolysis in Pure Water at Ampere-Level Current Density and 1000 h Stability via a Rapid-Transport Fixed-Charge Interface**

*Qiqi Wan, Gang Zhu, Wenxing Jiang, Yingying Liu, Jie Gui, Chi Xu, Ji Chen, Xiaodong Zhuang,* and Changchun Ke**

Q. Wan, W. Jiang, C. Ke

Institute of Fuel Cells

School of Mechanical Engineering

Shanghai Jiao Tong University

800 Dongchuan Rd, Shanghai 200240, P. R. China

1. mail: kechangchun@sjtu.edu.cn

G. Zhu

Wuhan Institute of Marine Electric Propulsion

Wuhan 430064, P. R. China

1. Liu, J. Gui, C. Xu, J. Chen

Shanghai Phoenix Technology Co., Ltd

55 Bixi Rd, Shanghai 200245, P. R. China

X. Zhuang
School of Chemistry and Chemical Engineering

Shanghai Jiao Tong University

800 Dongchuan Rd., Shanghai 200240, P. R. China
E-mail: zhuang@sjtu.edu.cn

**Experimental**

**Materials**

IrO_2_ nanopowders (Zhongke Jinyan), carbon blacks (Vulcan XC-72, Cabot), Ag nanopowders (≈ 200 nm, 99.99 % metal basis), Nafion^®^ resin solution (5 wt% in water/1-propanol, Dupont), PiperION^TM^ anion-exchange ionomer dispersion (5 wt%, C5-HCO_3_), poly(diallyldimethylammonium) chloride (PDDA) solution (20 wt % in water, Adamas), absolute ethanol (Sinopharm Chemical Reagent), anion exchange membrane (PiperION-A40, Versogen), Ti felt (0.25 mm, Bekaert) and carbon paper (E20H, Freudenberg) were used as received. Ultrapure water (18.2 MΩ·cm, 25 ℃) used in all experiments was produced by a Millipore Milli-Q system.

**Electrode Fabrication**

For the anode, a catalyst ink was prepared by dispersing IrO_2_ (50 mg) and Nafion^®^ solution (120 µL, 5 wt %) in ethanol (30 mL), followed by ultrasonication until homogeneous. The suspension was then deposited onto a 6 cm × 6 cm Ti felt by an ultrasonic spraying equipment, yielding an IrO_2_ loading of ~1.0 mg cm^-2^.

Ag-based cathode GDEs were fabricated similarly. For the cathode, Ag nanoparticles (64 mg), XC-72 carbon (64 mg), PTFE particles (12.8 mg) or PiperION^TM^ anion-exchange ionomer dispersion (256 mg, 5 wt %) or PDDA solution (64 mg, 20 wt %), and ethanol (60 mL) were mixed and ultrasonicated, then spray-coated onto carbon paper with the substrate held at 60 ℃, obtaining a conventional catalytic interface with dense polymer. Specifically, the interface of PDDA is denoted as DFC-I in the work. The loading of Ag is set to 0.8 mg cm^-2^.

Electrodes with larger area were prepared by proportionally scaling the ink volume and spray area while maintaining the same catalyst loadings.

**Electrochemical Characterization**

CO_2_RR performance was evaluated in a zero-gap electrolyzer.^[1]^ The cell was assembled as follows: cathode end-plate with flow field, gasket, Ag-based cathode, PiperION-A40 anion-exchange membrane, gasket, Ti felt anode with IrO_2_, and anode end-plate with flow field, with each catalyst layer facing the membrane. The screws were tightened in a diagonal sequence to ensure uniform compression. The zero-gap electrolyzers of 80 cm^2^ and 320 cm^2^ used the same structure and assembly sequence.

During operation, dry CO_2_ (100 sccm) was fed to the cathode inlet via a digital mass flow controller (Beijing Sevenstar). Ultrapure deionized water served as the anolyte and was circulated through the anode using a peristaltic pump. The cathode effluent first passed through a digital mass flowmeter to record the outlet flow rate and was then directed to a gas chromatograph (Shimadzu, GC 2014C) for compositional and concentration analysis. Electrochemical measurements for the 4 cm^2^ electrolyzer were performed on a Gamry Reference 5000 potentiostat. For the 80 and 320 cm^2^ electrolyzers, Kikusui power supplies (PWR401L and PWR2001L, respectively) provided galvanostatic control and data logging. All cell voltages reported in the figures are recorded without iR correction.

For the electrochemical reconstruction step, Ag-based electrodes were operated to an anodic current of 25 mA cm^-2^ for 40 s, immediately followed by standard cathodic CO_2_RR operation. Specifically, the PDDA-based interface formed through the reconstruction step is denoted as RTFC-I in the work.

Polarization curves were obtained by stepping the current density from 50 to 500 mA cm^-2^, holding each step for 10 min at 25 ℃ (terminated earlier if the cell voltage exceeded 6 V). After 9 min at each step, the cathode effluent was sampled and analyzed by GC.

The Faradaic efficiency (FE) was calculated as: $\text{FE}\text{ }\text{=}\frac{\text{c}\text{ × }\text{F}_{\text{v }}\text{× }\text{P}\text{ }\text{×}\text{ }\text{n}\text{ ×}\text{ }\text{F}}{\text{R }\text{×}\text{ }\text{T}\text{ }\text{×}\text{ }\text{I}}\text{×100\%}$. Where *c* is the mole fraction of the gaseous product as measured by GC, *F*_v_ is the gas flow rate at the cathode outlet (measured by mass flowmeter), *P* and *T* are the gas pressure and temperature at which *F*_v_ is defined, *n* is the electron number for the product, F is the Faraday constant, R is the ideal gas constant, and *I* is the applied current.

The CO partial current density in Figure 2b was obtained by recording the steady-state cell voltage at different current densities. At each steady state, the CO partial current density was calculated by multiplying the total current density by the FECO.

**Materials Characterization**

Scanning electron microscopy (SEM) and EDS elemental mapping were performed on a TESCAN MAGNA SEM equipped with an EDS detector. Transmission electron microscopy (TEM) and STEM-EDS mapping were conducted on a Talos F200X (Thermo Fisher Scientific) operated at 200 kV. CO_2_ adsorption isotherms were measured at 25 ℃ on a BELSORP-MAX (MicrotracBEL, Japan), and N_2_ adsorption-desorption isotherms on an ASAP 2460 (Micromeritics). X-ray photoelectron spectroscopy (XPS) spectra were acquired on an AXIS Ultra DLD (Shimadzu Corporation). Powder X-ray diffraction (XRD) patterns were collected on a D8 ADVANCE Da Vinci (Bruker).

***In situ* Electrochemical Measurements**

Differential electrochemical mass spectrometry (DEMS) was performed on Linglu Instrument (Shanghai) Co., Ltd., coupled to a potentiostat (Autolab PGSTAT302N), for real-time detection of gaseous products during CO_2_RR. A standard three-electrode configuration was employed: an Ag-based gas-diffusion electrode (GDE) as the working electrode, a Pt wire as the counter electrode, and an Ag/AgCl reference electrode. The electrolyte was CO_2_-saturated 0.1 M KHCO_3_ and CO_2_ was supplied at 20 mL min^-1^ via a mass flow controller. Potentials are recorded versus the reversible hydrogen electrode (vs. RHE). During measurements, the potential of working electrode was swept from 0.00 to -1.30 V at 5 mV s^-1^. Mass spectrometric signals were recorded continuously for the relevant channels (e.g., m/z=28 for CO and m/z=2 for H_2_).

*In situ* FTIR spectra were collected on a Thermo Scientific™ Nicolet iS50 FTIR spectrometer. The Ag-based catalyst inks were sprayed onto an Au substrate to serve as the working electrode. A Pt wire and an Ag/AgCl reference electrode were used as the counter and reference electrodes, respectively. The electrolyte was CO_2_-saturated 0.1 M KHCO_3_ with CO_2_ supplied at 20 mL min^-1^ via a mass flow controller. Spectra were recorded with a time resolution of ~19 s per spectrum. Background spectra were first acquired at open-circuit potential in CO_2_-saturated electrolyte. In the main text, the spectra retain the original CO_2_ background feature in order to compare the initial CO_2_ adsorption capability of different interfaces. For comparison, an additional set of background-subtracted spectra was further presented by using the spectrum of open-circuit potential as the reference, so that the onset of local CO_2_ consumption during CO_2_RR could be more clearly identified. The electrode potential was applied from -0.1 V to -2.0 V vs. RHE, with a dwell time of 60 s at each potential.

**DFT Calculations**

Geometry optimizations and electronic structure calculations for Ag surfaces and the cation-modified models were performed with the Vienna *ab initio* simulation package (VASP).^[2]^ Exchange-correlation effects were treated within the gradient approximation (GGA) using the PBE functional,^[3]^ and long-range dispersion was included via the DFT-D3 scheme.^[4]^ The plane-wave cutoff energy was set to 400 eV. The Gamma point in the Brillouin-zone was chosen for integration. Electronic self-consistency was converged to 10^-5^ eV in the iteration solution of Kohn-Sham equation, and ionic relaxations proceeded until the residual forces on each atom were below 0.05 eV Å^-1^.

The CO_2_-to-CO pathway was modeled as two proton–electron transfer steps:

CO_2_ + H^+^ + e^-^ → *COOH

*COOH + H^+^ + e^-^ → *CO + H_2_O

*CO → CO + *

where * represents a vacant surface catalytic active site.

Gibbs free energies were calculated using the following equation:

G = E_DFT_ + E_ZPE_ - TS

where G is the Gibbs free energy, E_DFT_ is the DFT total energy, E_ZPE_ is the zero point energy, S is the entropy, and T is room temperature (298 K).

**Supplementary Figures**


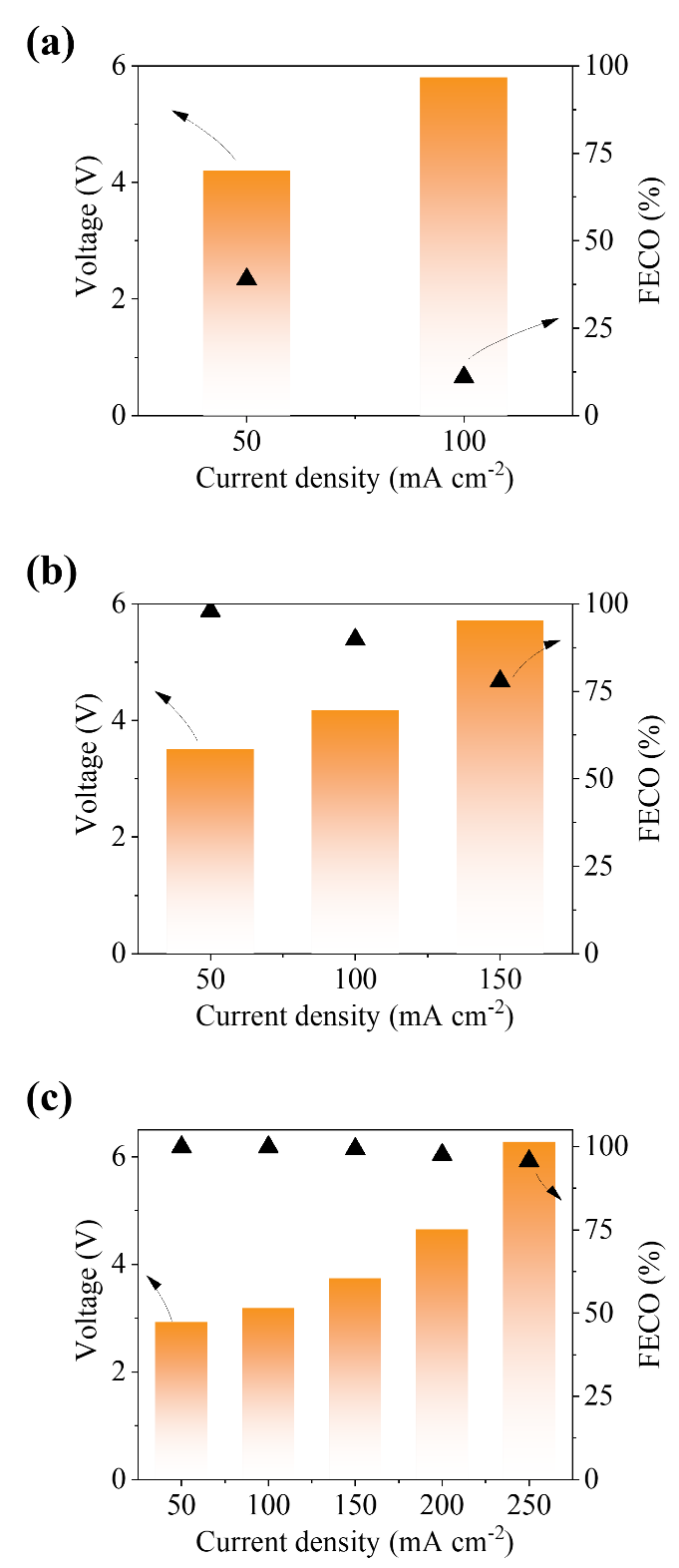


**Figure S1.** Cell voltage and FECO as a function of current density at 25 ℃ for the a) PTFE, b) PiperION, and c) PDDA electrode.


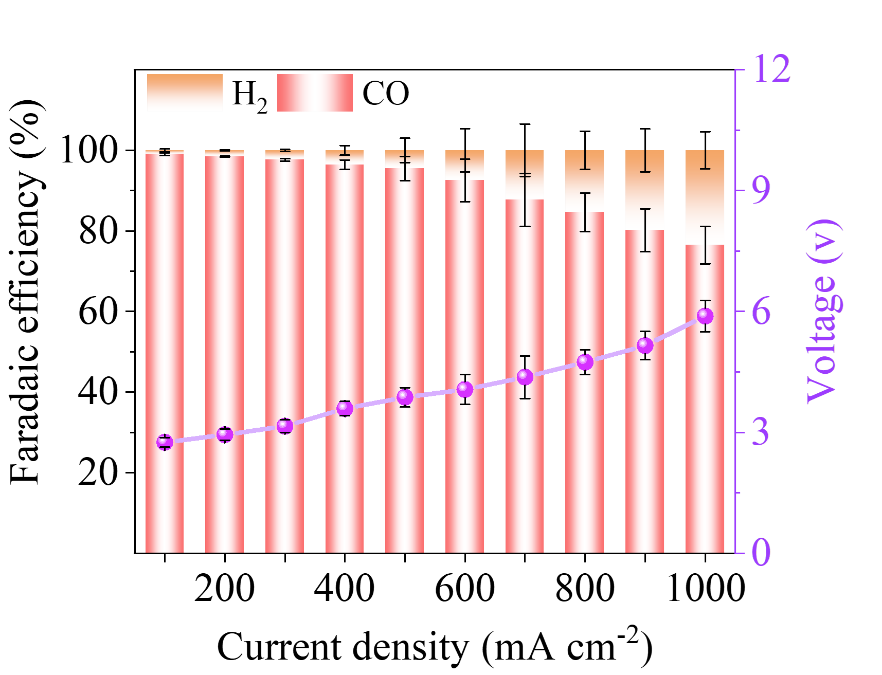


**Figure S2.** Electrochemical performance of the RTFC-I for CO_2_RR in pure water. FECO and cell voltage as a function of current density at 60 ℃.


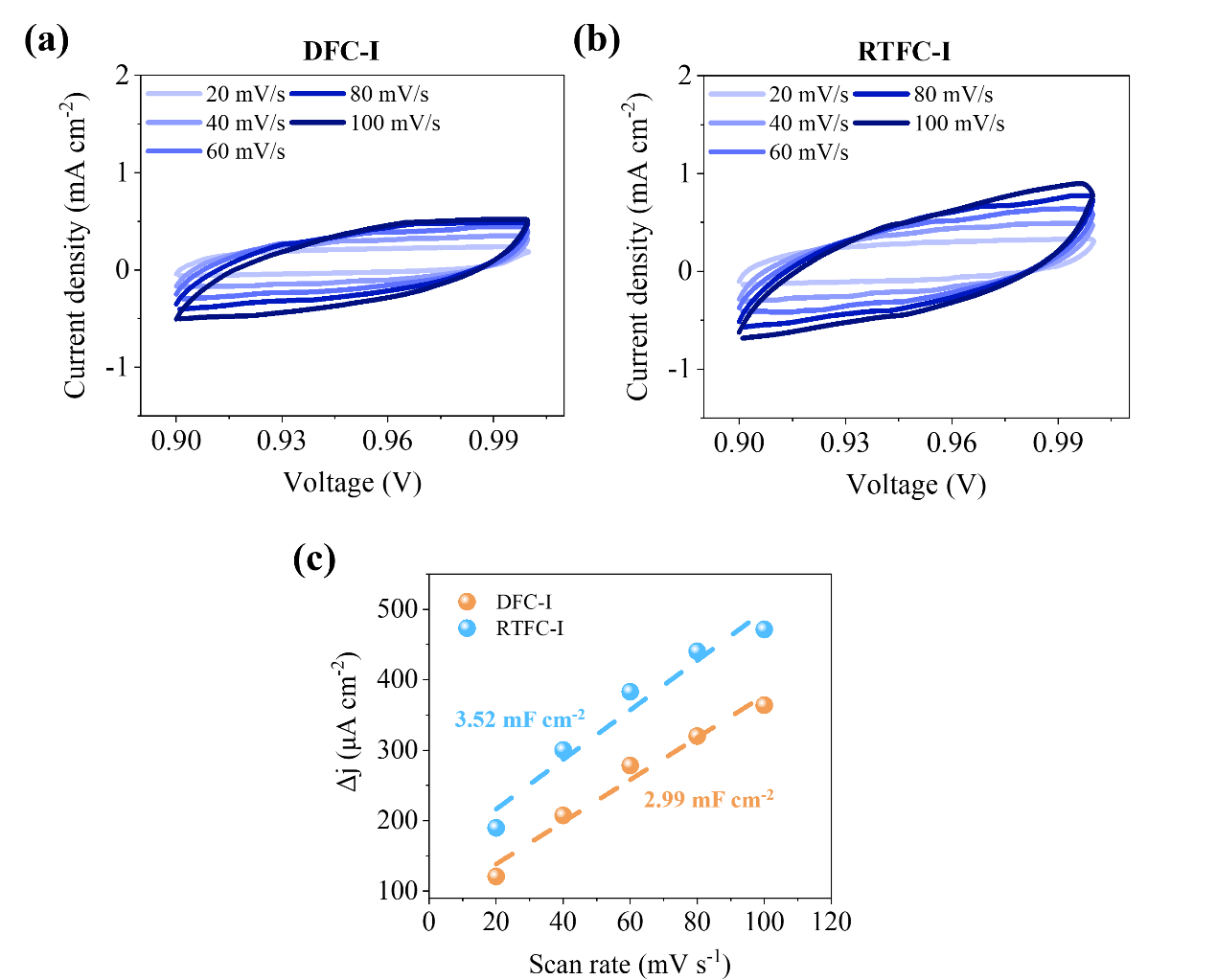


**Figure S3.** Double-layer capacitance measurements. Cyclic voltammograms recorded at various scan rates within a non-Faradaic potential window for a) DFC-I and b) RTFC-I. c) A plot of Δj at a fixed potential versus scan rate; double-layer capacitance increases from 2.99 to 3.52 mF cm^-2^, indicating an enlarged electrochemically active surface area.


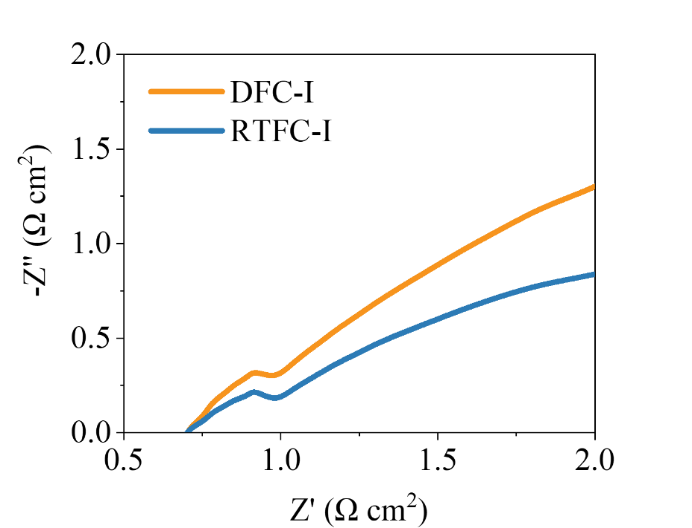


**Figure S4.** Electrochemical impedance spectroscopy (EIS) of DFC-I and RTFC-I.


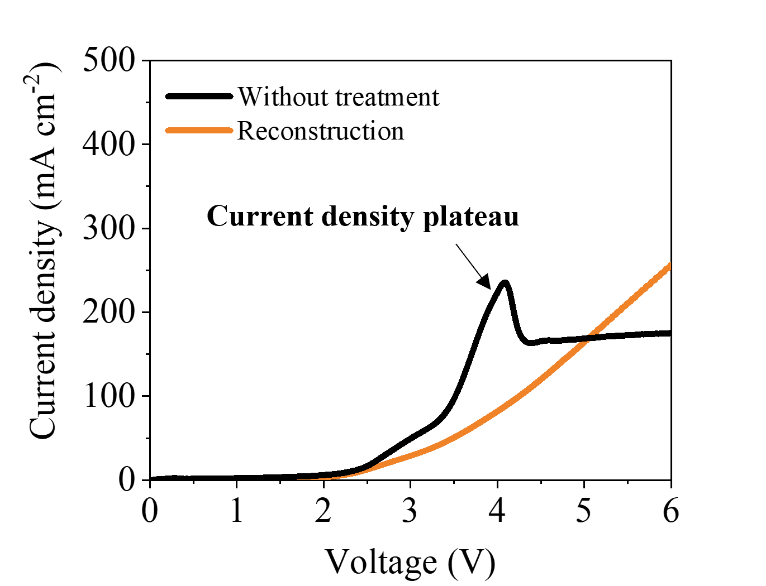


**Figure S5.** LSV of the PiperION-based electrode; the current plateau disappears after reconstruction.


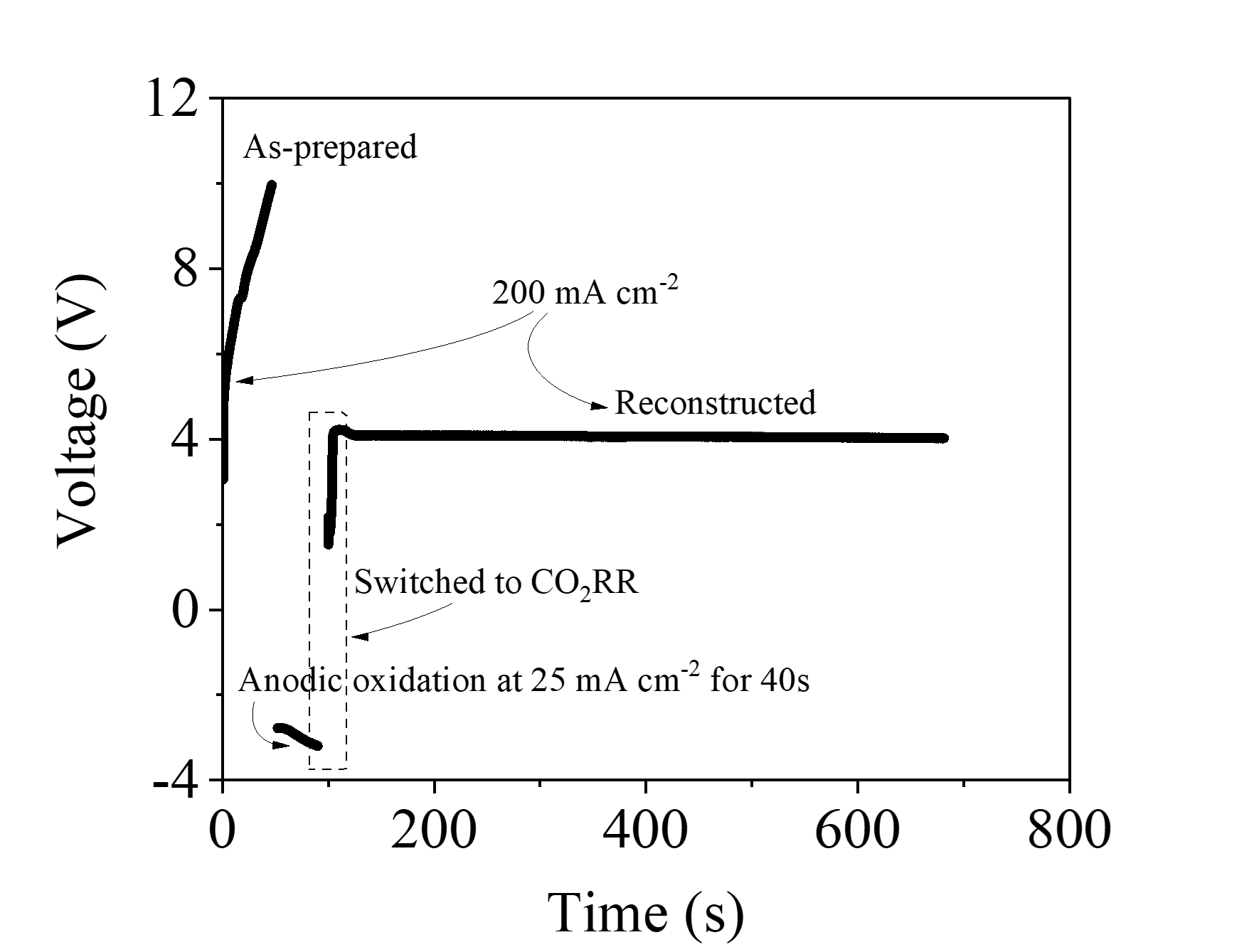


**Figure S6.** Galvanostatic performance at 200 mA cm^-2^ for the PiperION-based electrode before and after reconstruction. The as-prepared electrode failed to sustain operation at 200 mA cm^-2^, whereas the reconstructed electrode maintained the same current at a stable cell voltage of 4.04 V.


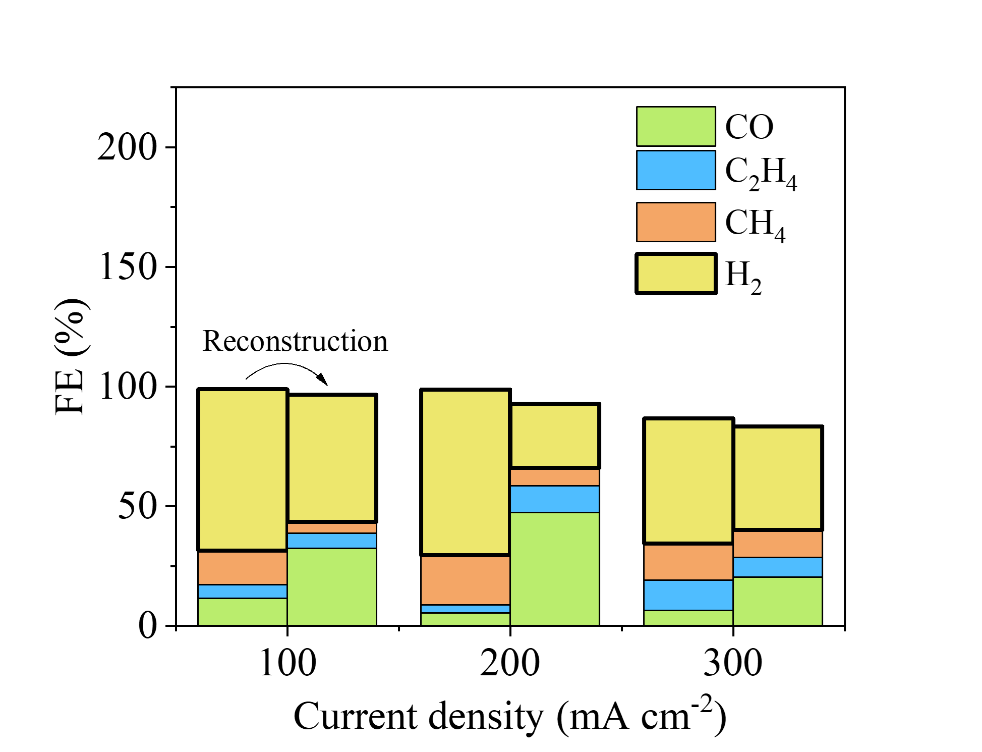


**Figure S7.** Product distribution of the PDDA-based Cu electrode before and after reconstruction at current densities of 100-300 mA cm^-2^. After reconstruction, the electrode shows increased formation of CO and C_2_H_4_ together with suppressed HER.


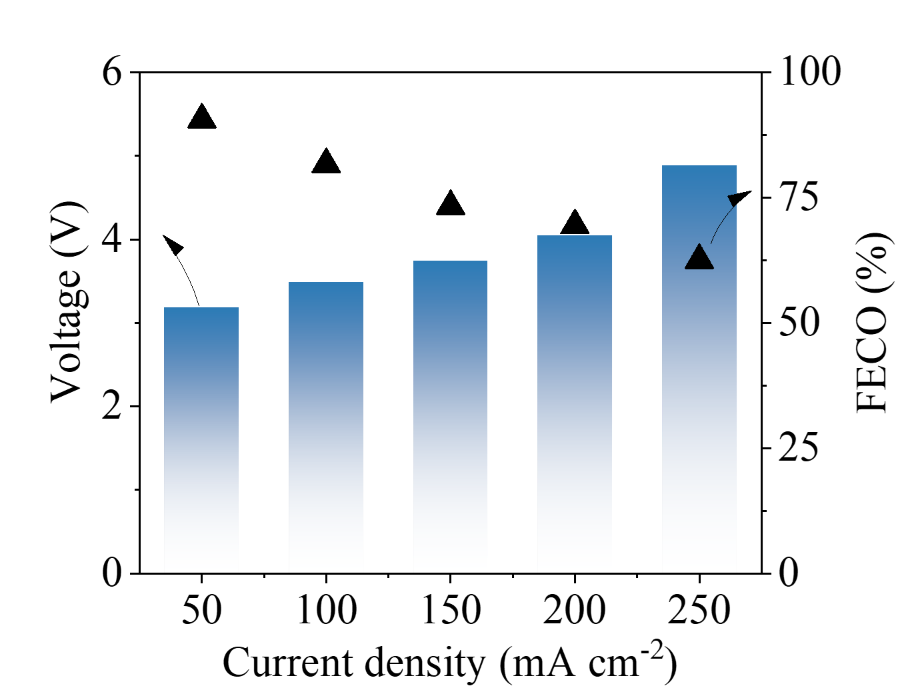


**Figure S8.** Cell voltage and FECO as a function of current density at 25 ℃ for the PiperION-based electrode with nanochannels.


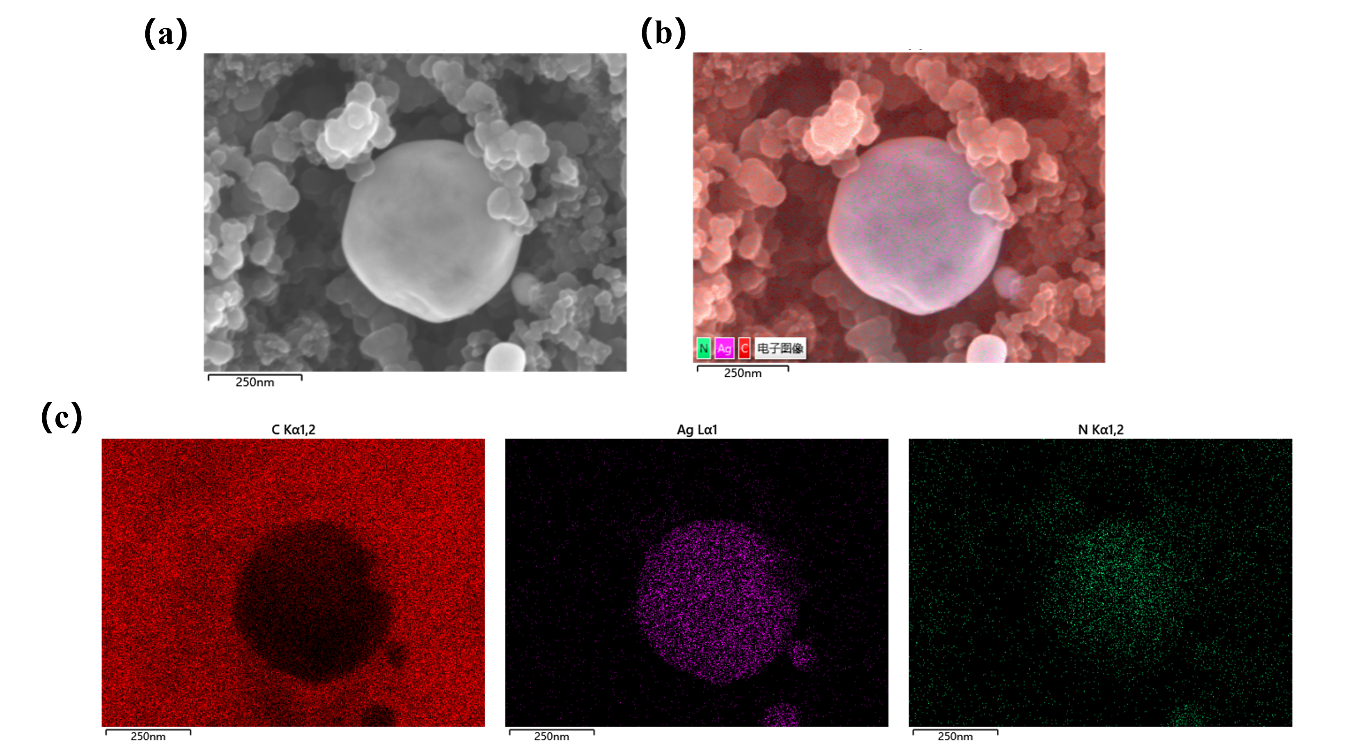


**Figure S9.** SEM morphology and EDS elemental mapping of DFC-I. a) Surface morphology. b) EDS elemental mapping showing the co-distribution of C, N (from PDDA) and Ag. c) EDS maps for N, Ag, and C (as labeled), displaying their respective spatial distributions across the electrode surface.


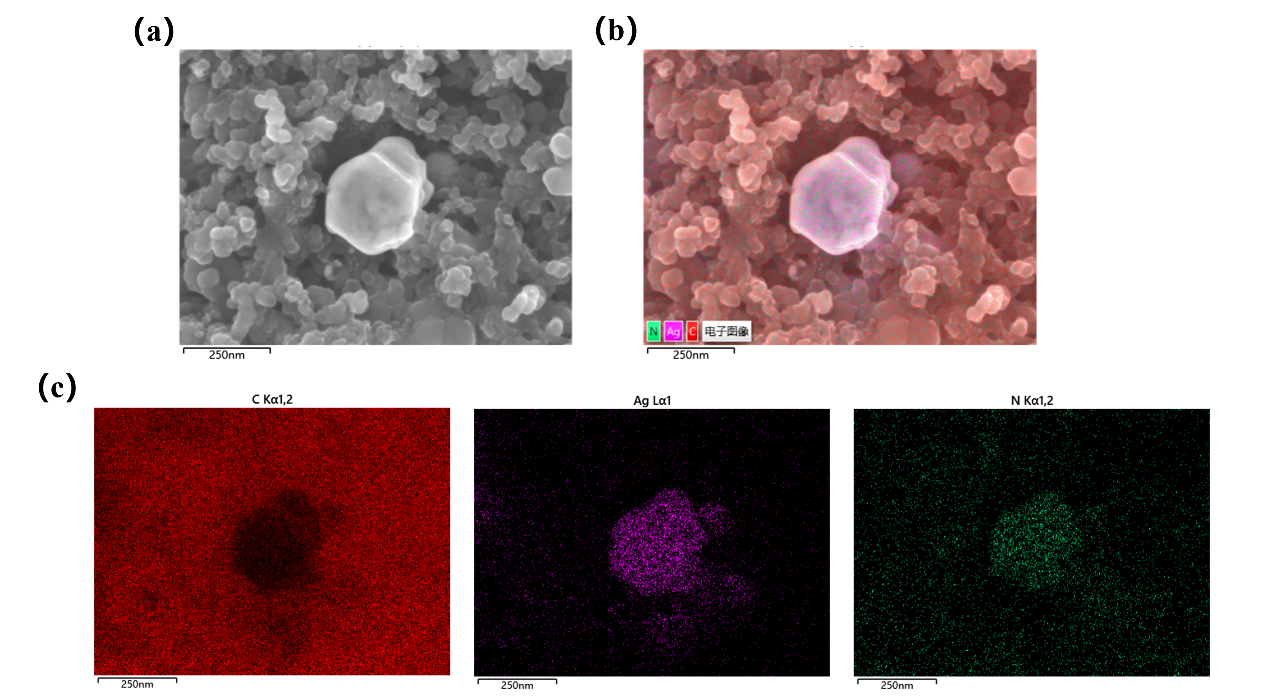


**Figure S10.** SEM morphology and EDS elemental mapping of RTFC-I. a) Surface morphology. b) EDS elemental mapping showing the co-distribution of C, N (from PDDA) and Ag. c) EDS maps for N, Ag, and C (as labeled), displaying their respective spatial distributions across the electrode surface.


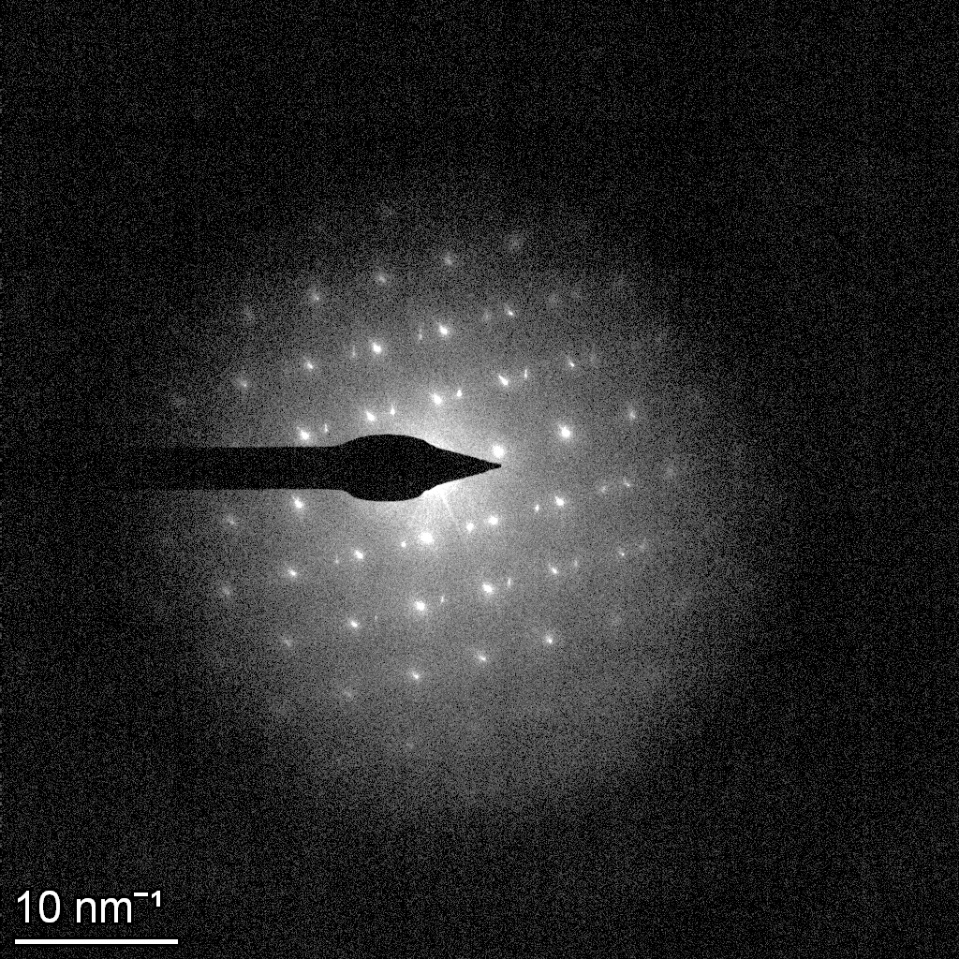


**Figure S11.** Selected-area electron diffraction (SAED) of DFC-I.


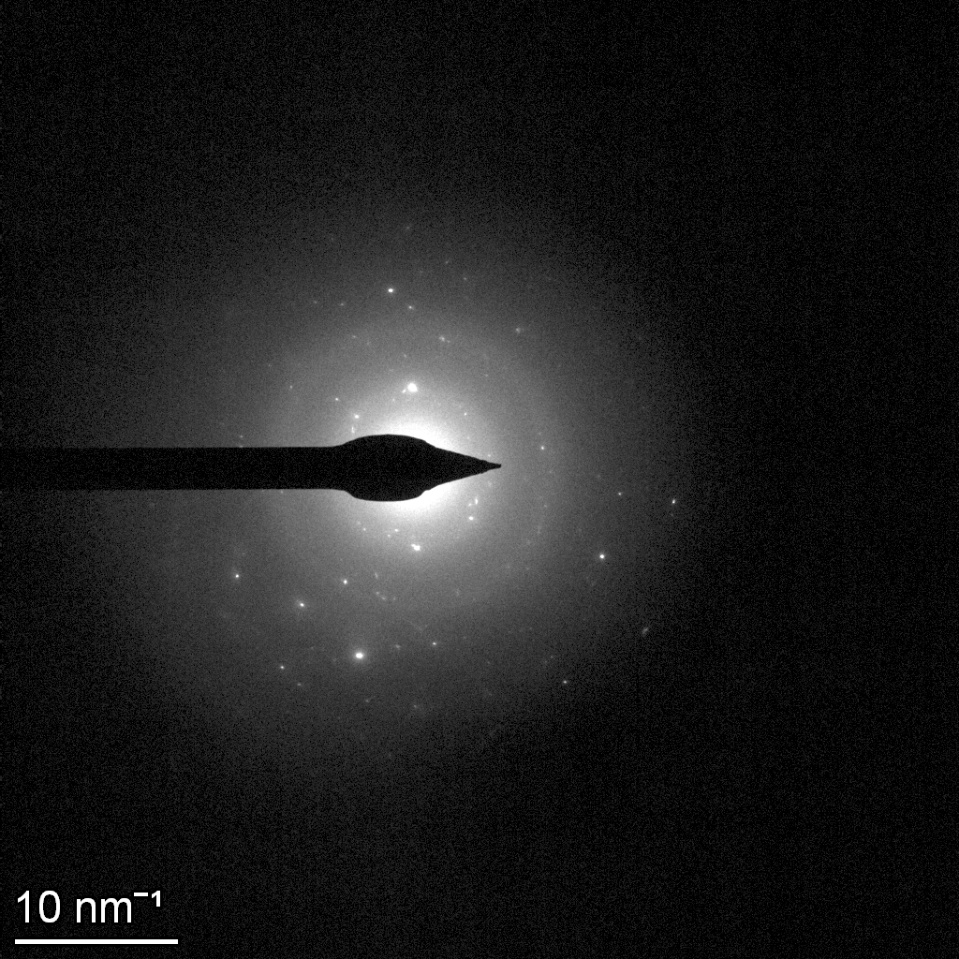


**Figure S12.** SAED of a representative Ag particle of RTFC-I, showing a set of concentric rings.


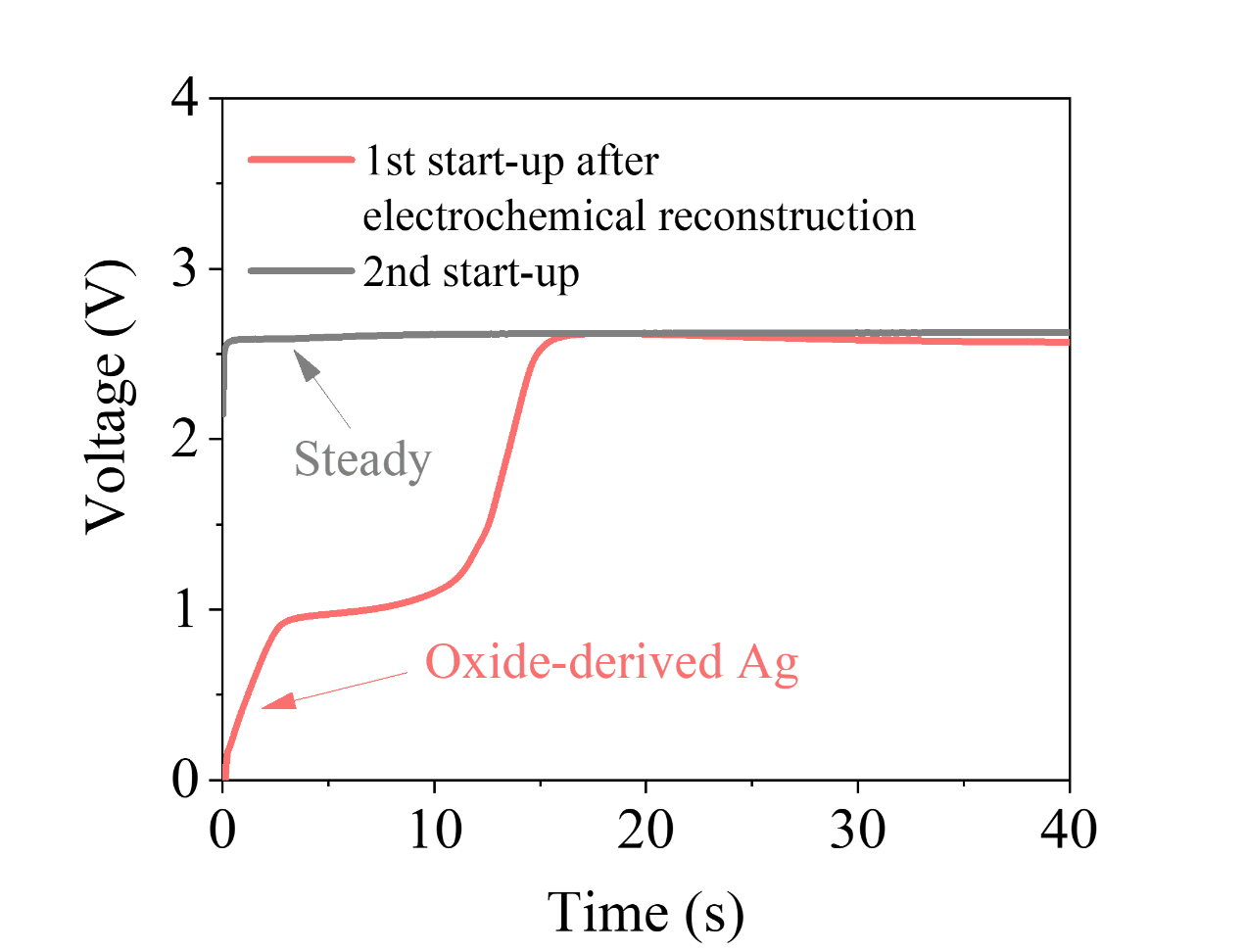


**Figure S13.** Transient signature of oxide reduction during the electrochemical reconstruction. After an anodic pulse at 25 mA cm^-2^ for 40 s, the electrolyzer was immediately switched to standard cathodic CO_2_RR at 50 mA cm^-2^. The cell voltage shows an initial low voltage well below the typical onset for water electrolysis/CO_2_RR (~0-18 s), indicating rapid reduction of the as-formed oxide. Subsequently, the voltage rises directly to the steady CO_2_RR operating value, evidencing that the oxide has been reduced.


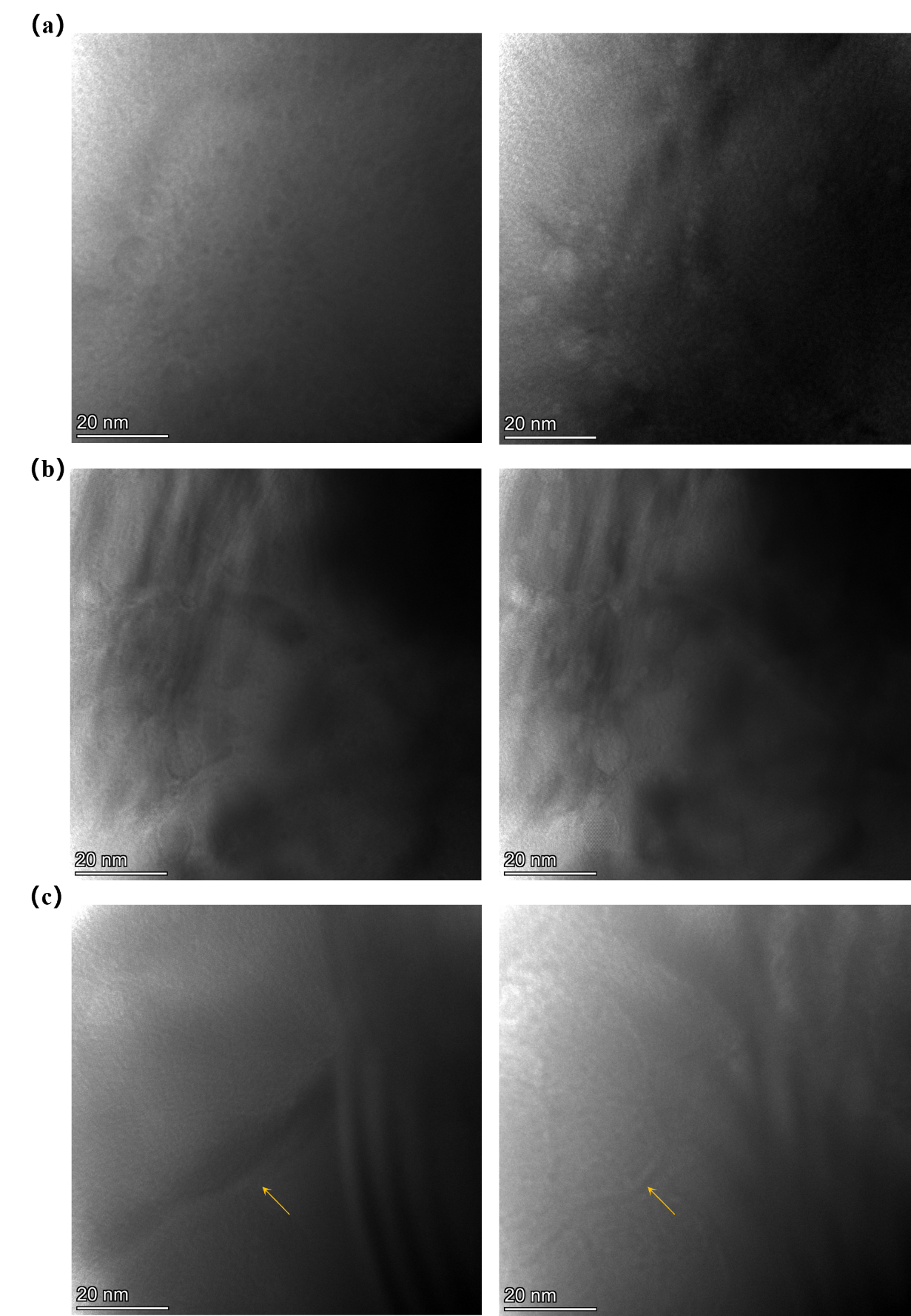


**Figure S14.** HRTEM images with Fresnel contrast inversion. The reversal of contrast from over-focus (left) to under-focus (right) confirms the presence of a,b) true cavities and c) surface cracks.


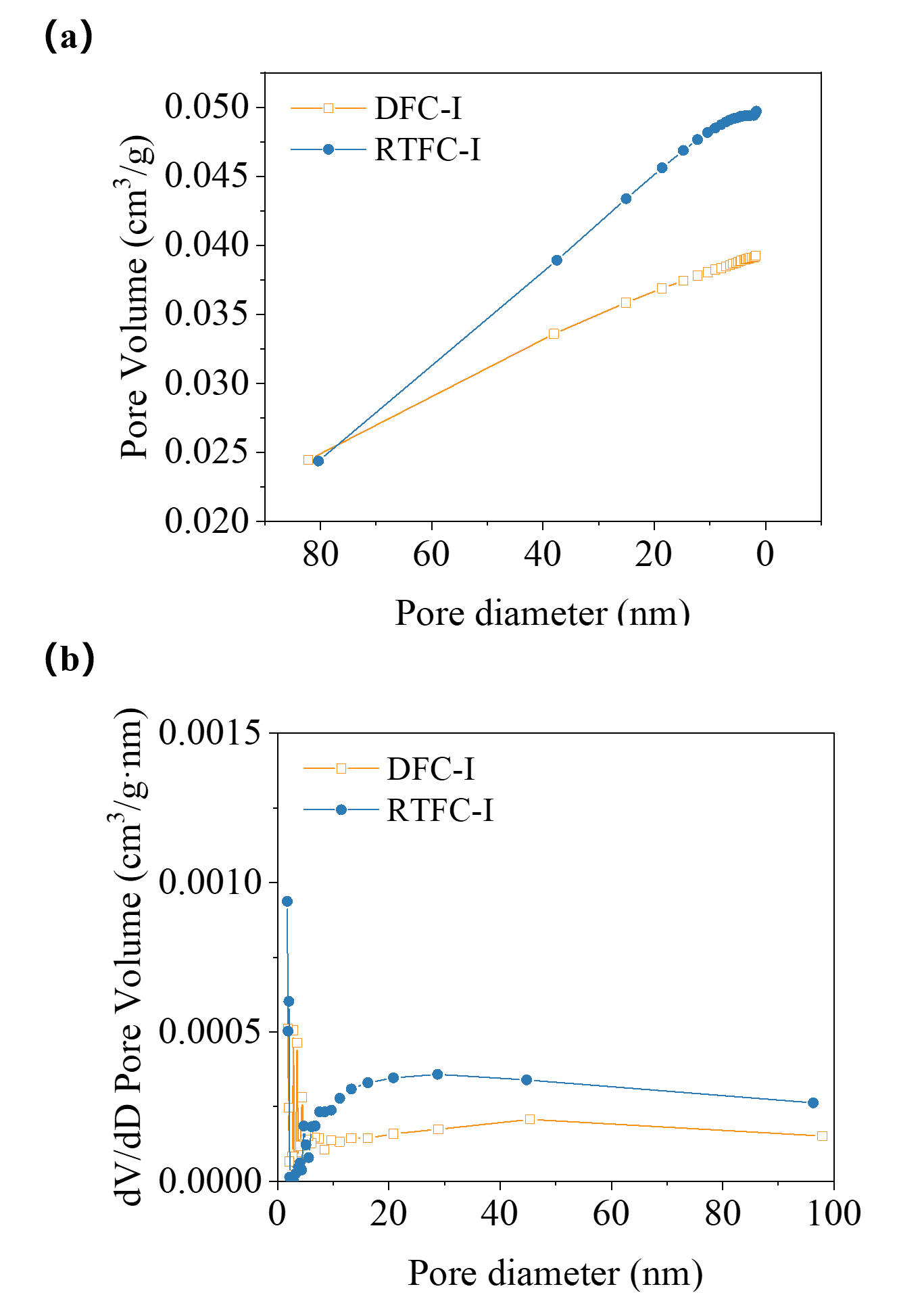


**Figure S15.** Pore-size distributions. a) Pore volume versus pore diameter. b) Differential pore volume versus pore diameter. It shows a clear increase in nanopore volume of RTFC-I, indicating the development of a more nanoporous morphology.


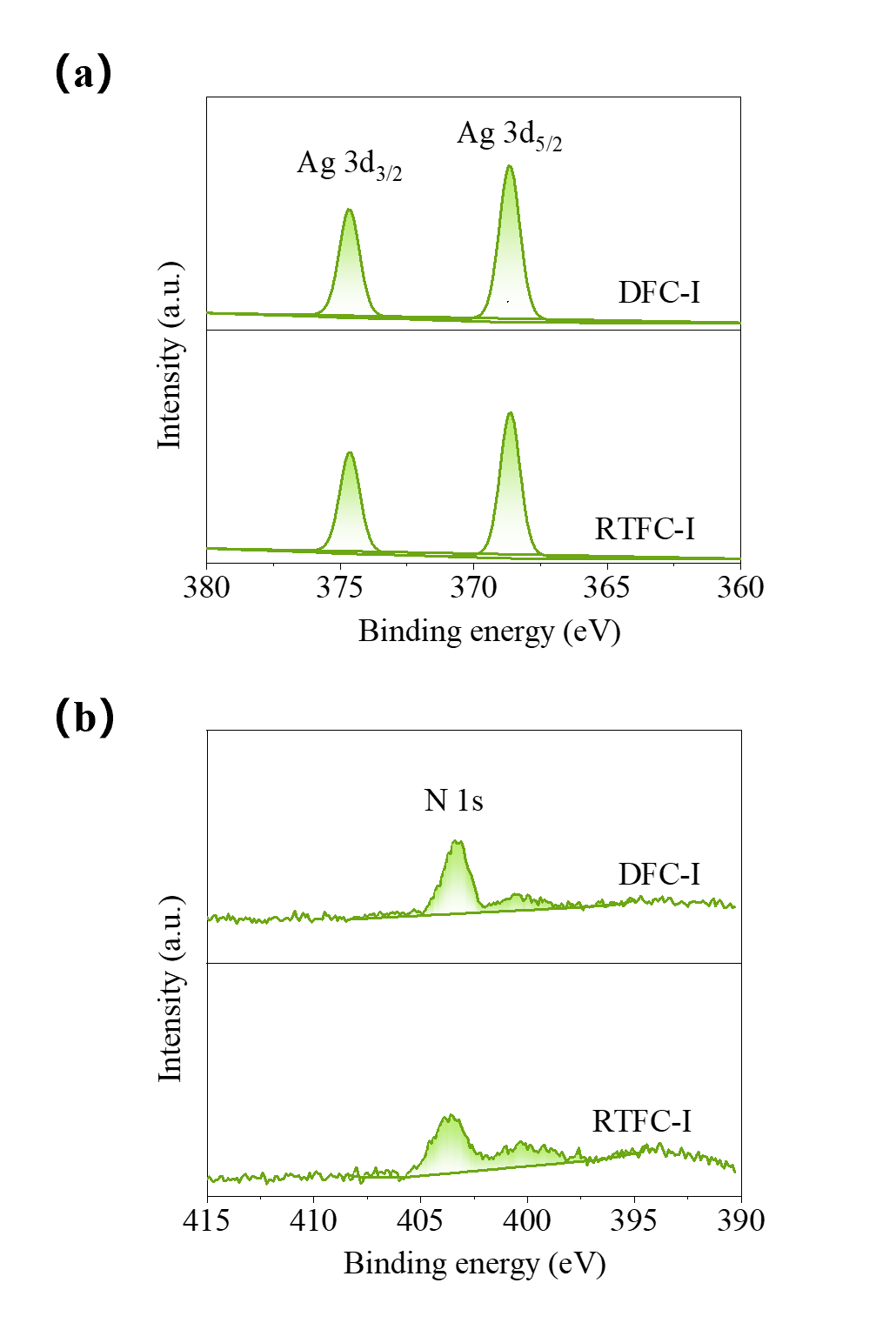


**Figure S16.** XPS spectra of the a) Ag 3d and b) N 1s for DFC-I and RTFC-I.


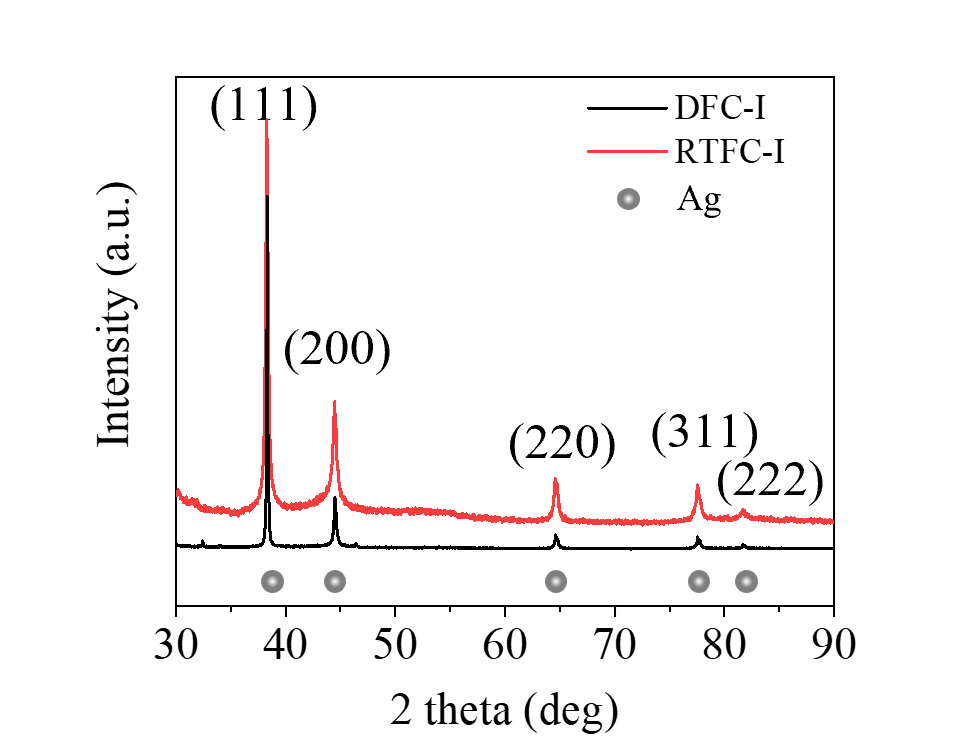


**Figure S17.** XRD patterns of DFC-I and RTFC-I.


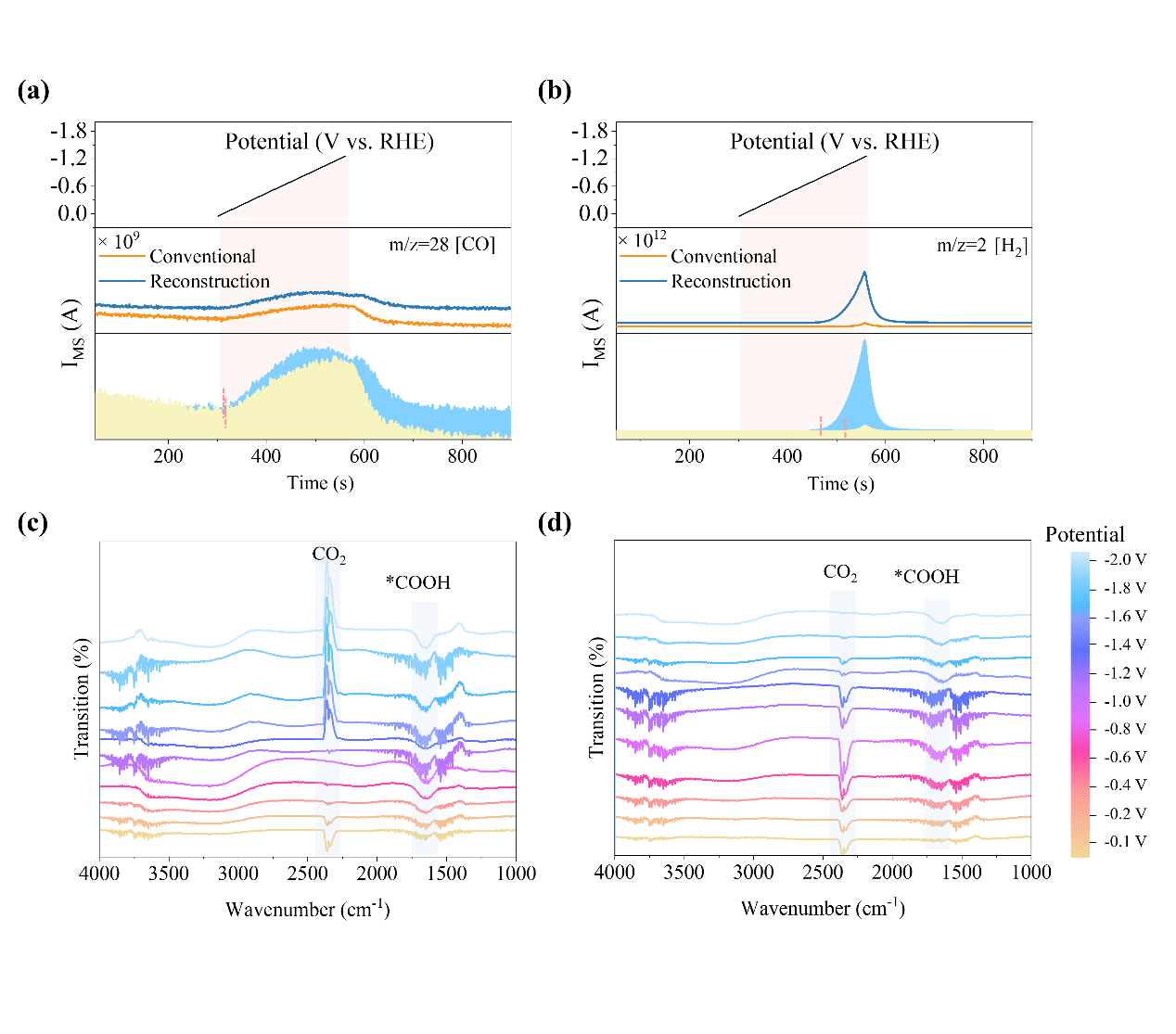


**Figure S18*.*** *In situ* spectroscopic analysis on the PiperION-based Electrode. a,b) DEMS results, tracking the evolution of CO and H_2_. For each subplot, the top panel shows the applied potential, the middle panel displays the corresponding ion current signal for the product, and the bottom panel provides a magnified view of the low-signal region. c,d) *In situ* FTIR spectra recorded at various applied potentials for the PiperION electrode before and after electrochemical reconstruction.


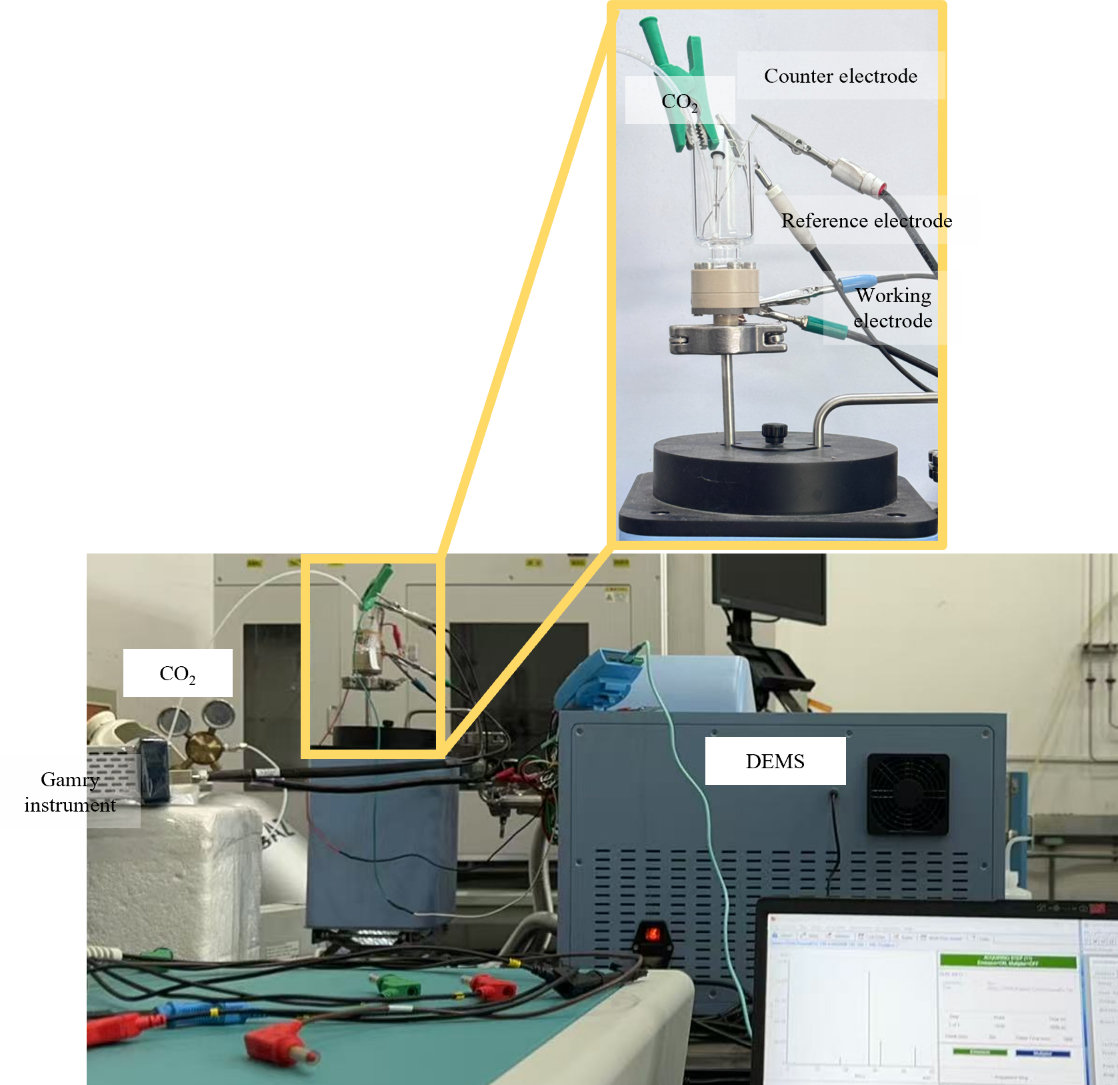


**Figure S19.** Schematic of the *operando* DEMS setup.


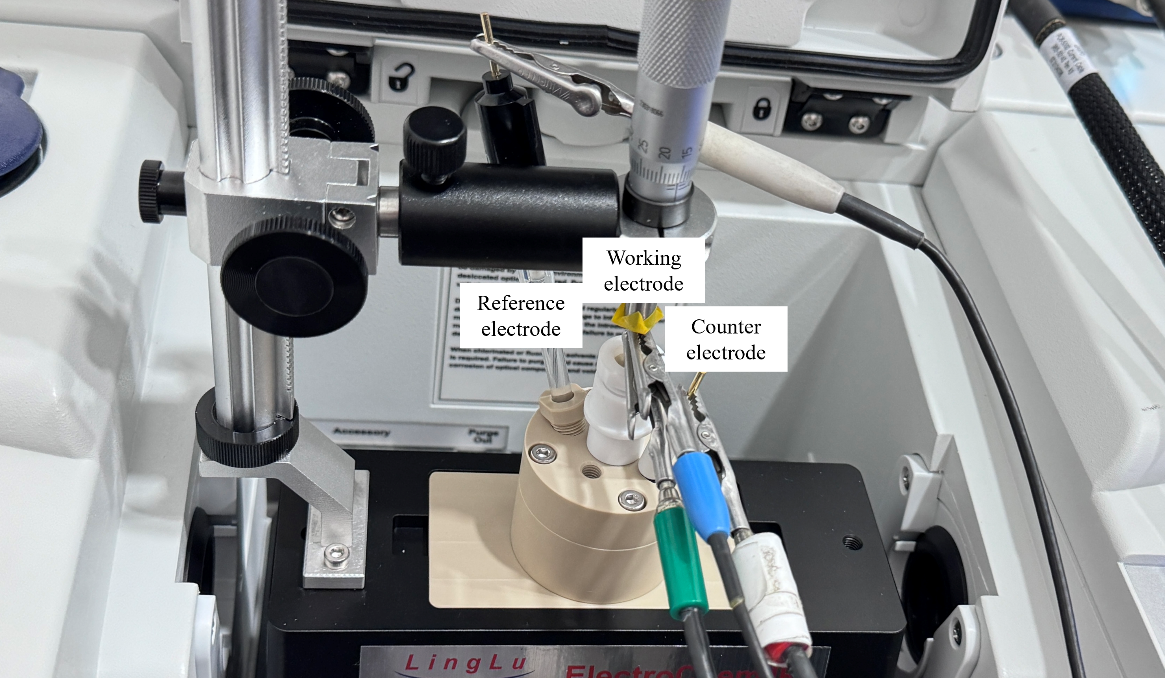


**Figure S20.** Schematic of the *in situ* FTIR setup.


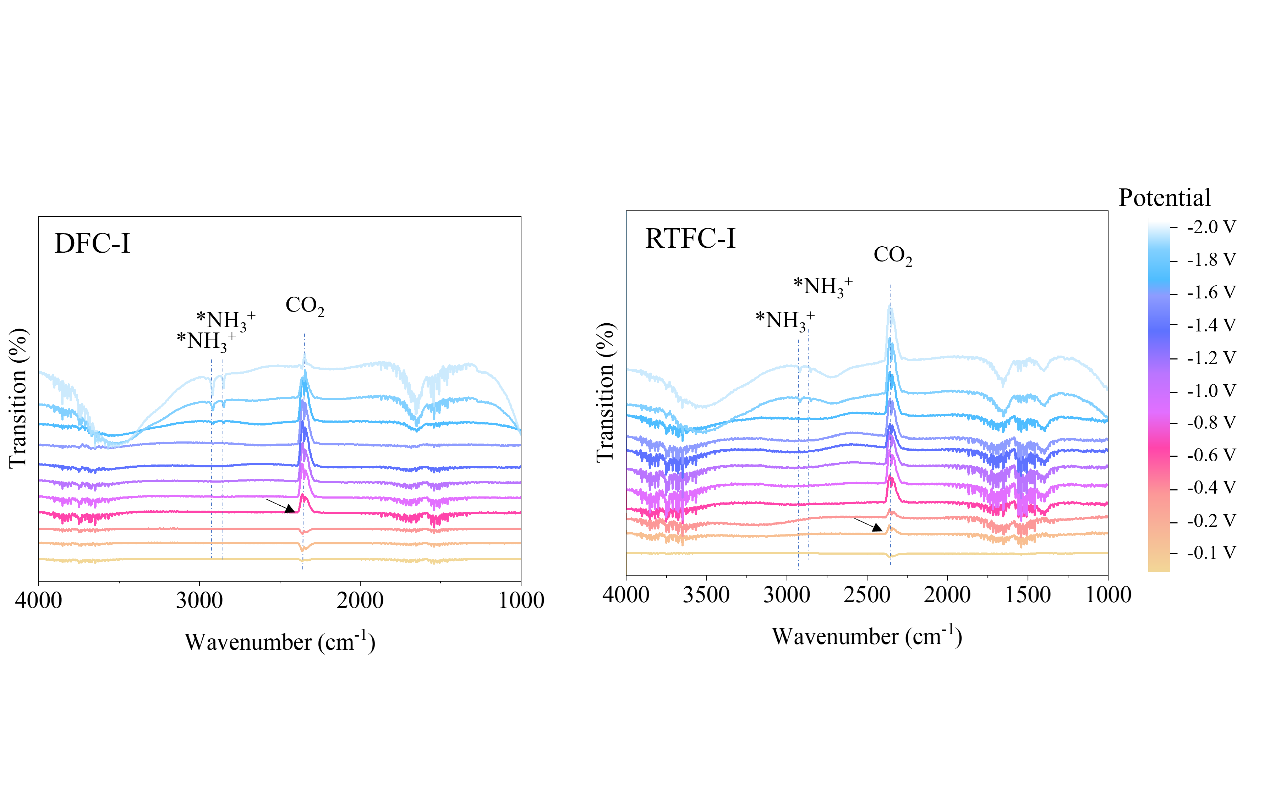


**Figure S21.** Background-subtracted *in situ* FTIR spectra of DFC-I and RTFC-I.

**
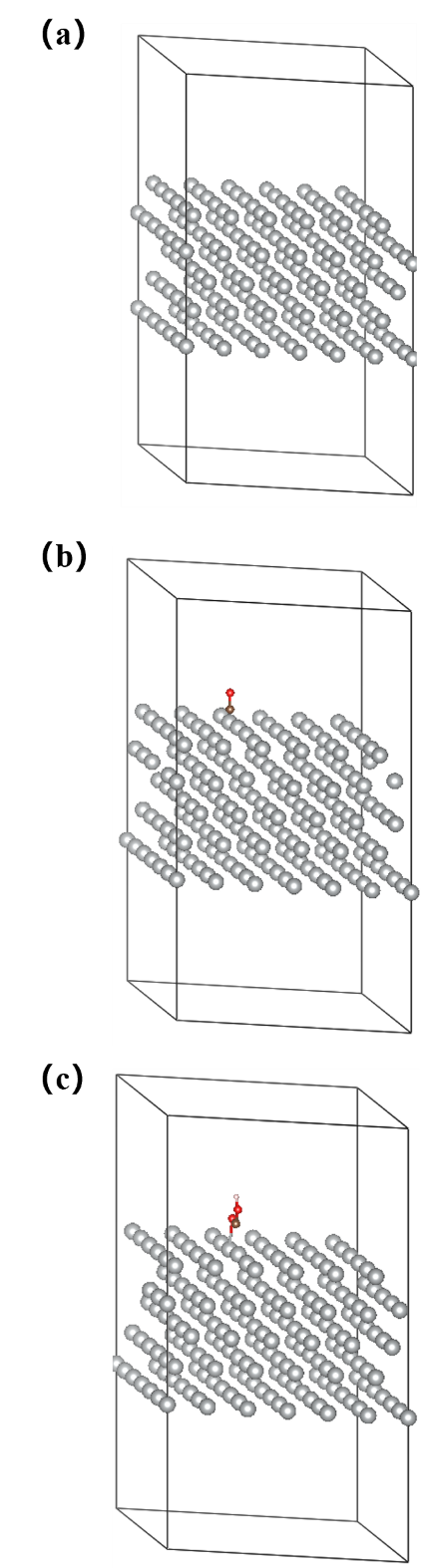
**

**Figure S22.** Optimized geometries of a) bare Ag(111), b) CO* on Ag(111), and c) *COOH on Ag(111).

**
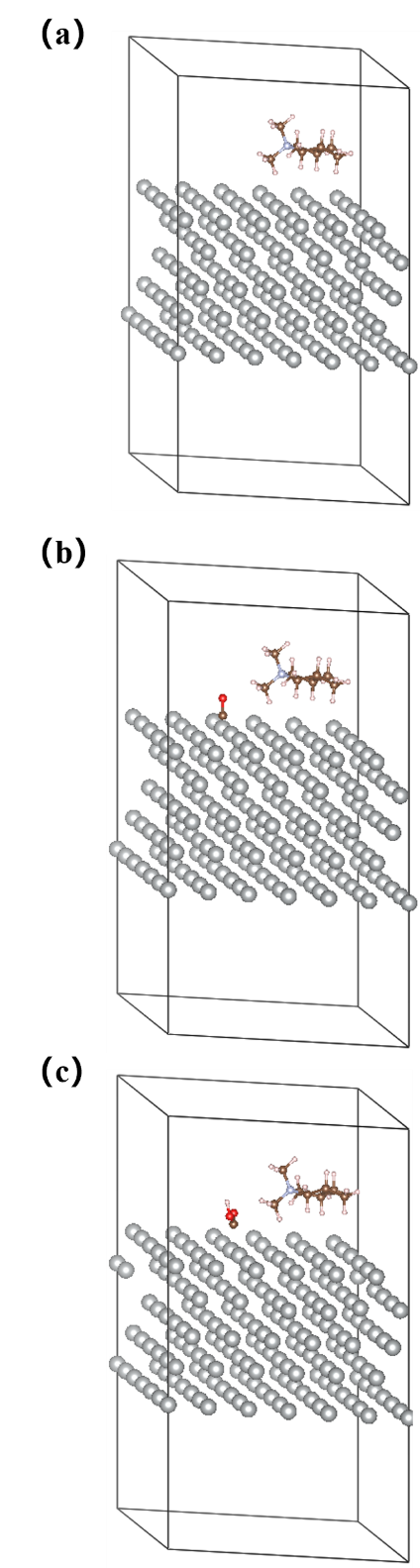
**

**Figure S23.** Optimized geometries of a) cation-based Ag(111), b) CO* on cation-based Ag(111), and c) *COOH on cation-based Ag(111).

**
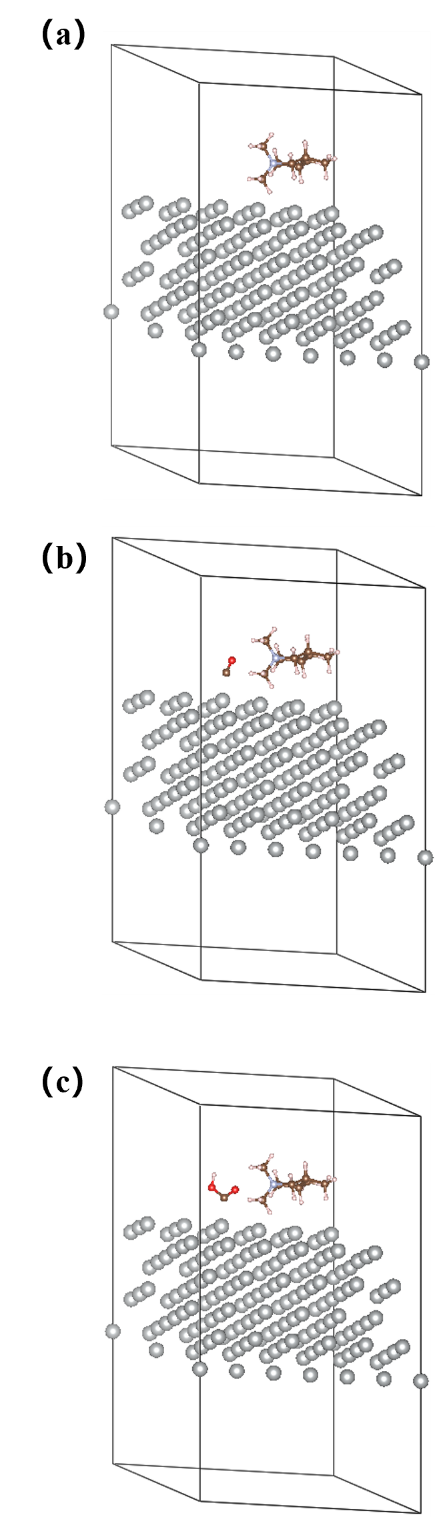
**

**Figure S24.** Optimized geometries of a) cation-based Ag(211), b) CO* on cation-based Ag(211), and c) *COOH on cation-based Ag(211).


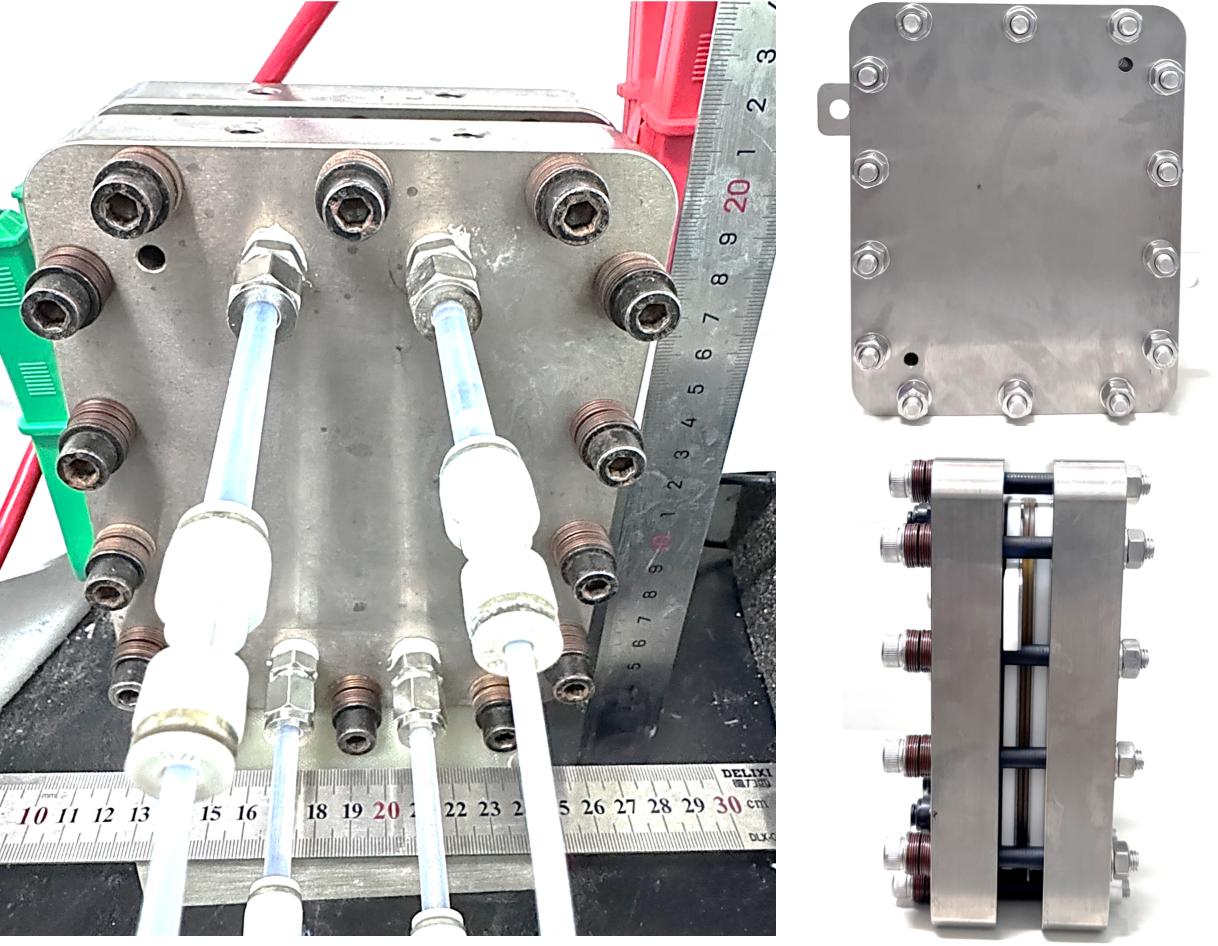


**Figure S25.** Photos of the 80 cm^2^ zero-gap electrolyzer.


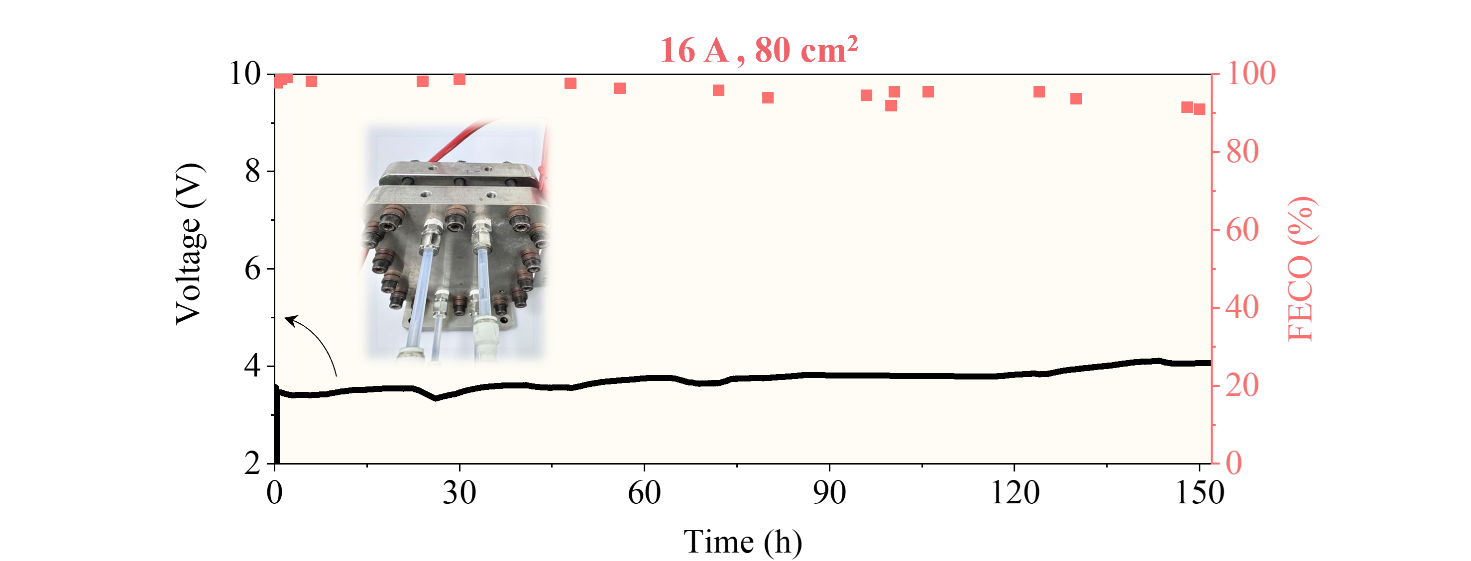


**Figure S26.** Durability test in a scaled-up 80 cm^2^ electrolyzer at 200 mA  cm^-2^ and 25 ℃.

**
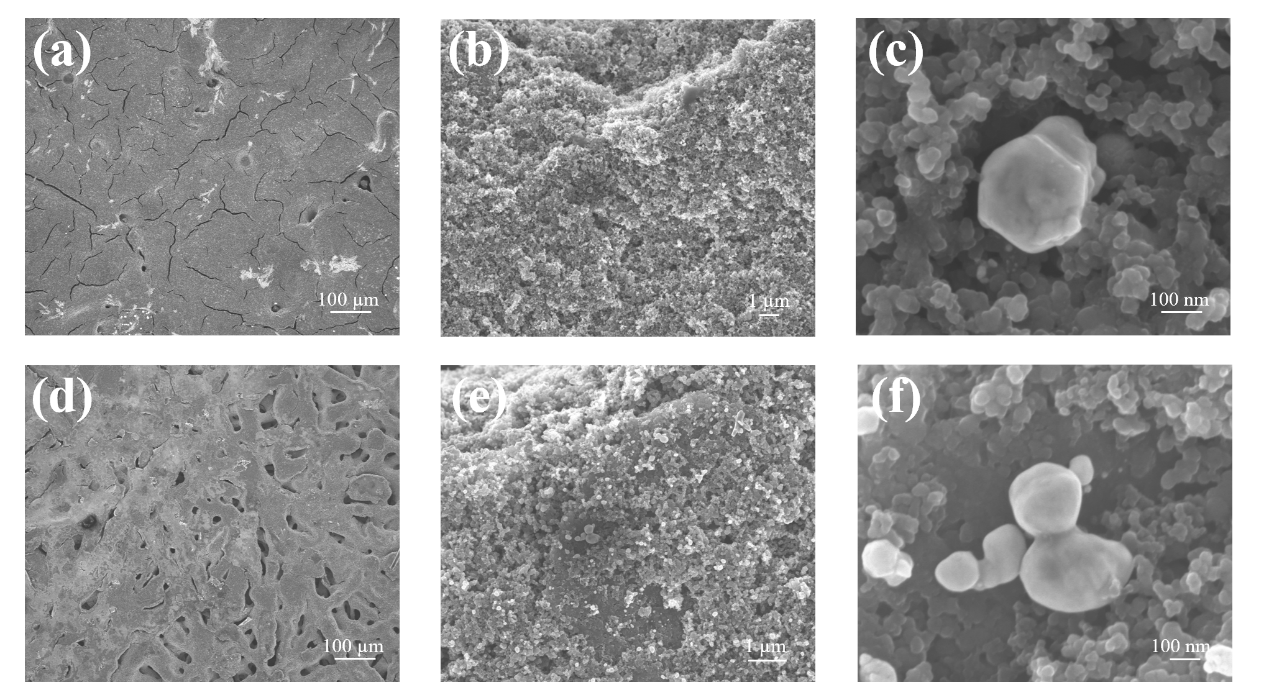
**

**Figure S27.** SEM images of RTFC-I at different magnifications before and after the durability test: a-c) pre-stability and d-f) post-stability.


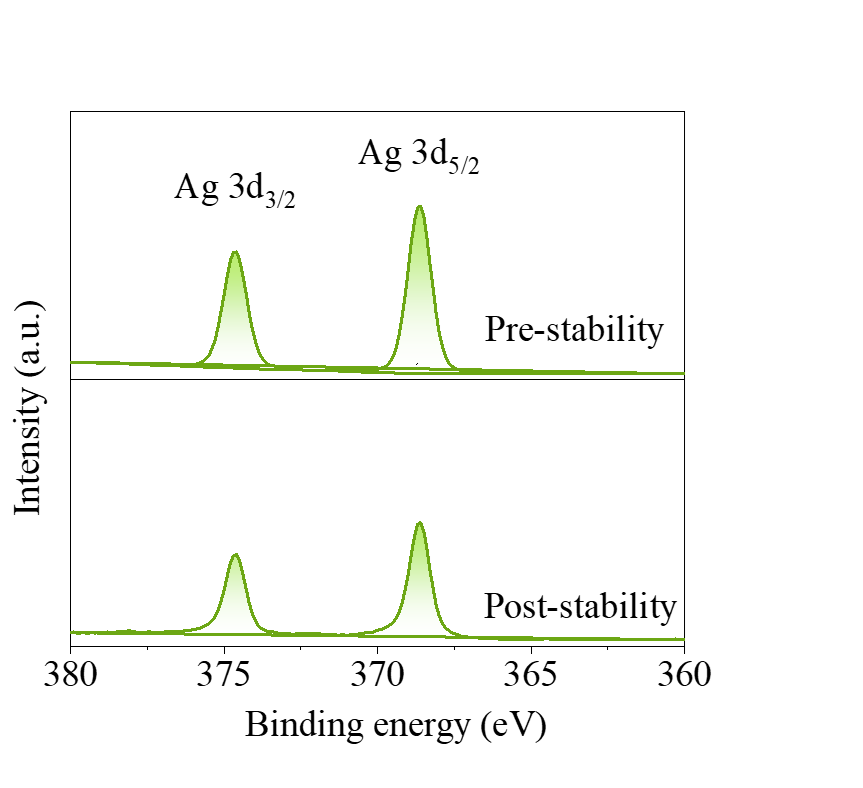


**Figure S28.** XPS spectra of the Ag 3d for RTFC-I before and after durability test.


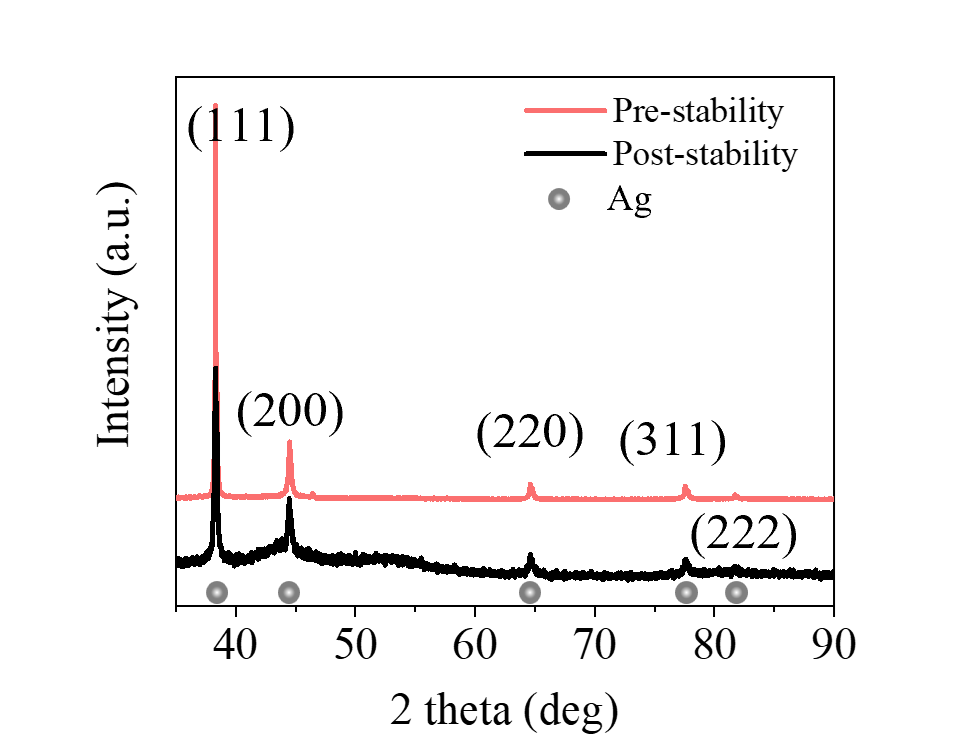


**Figure S29.** XRD patterns of RTFC-I before and after durability test.

**
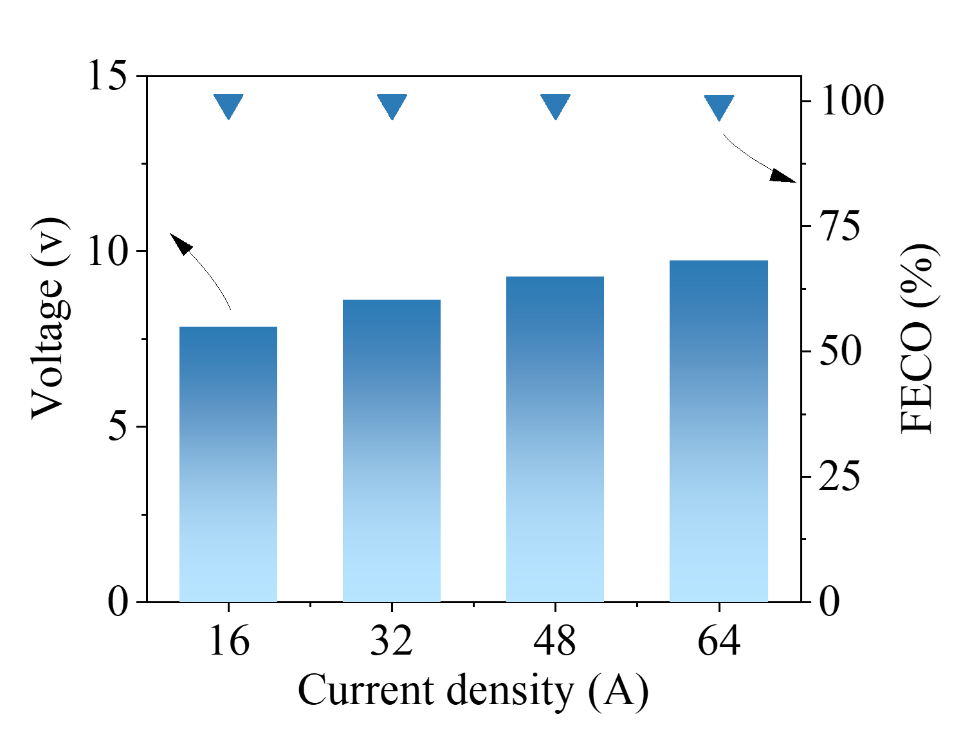
**

**Figure S30.** Total voltage and FECO at various currents for the three-cell stack of RTFC-I (series connection; 320 cm^2^ per cell).

**
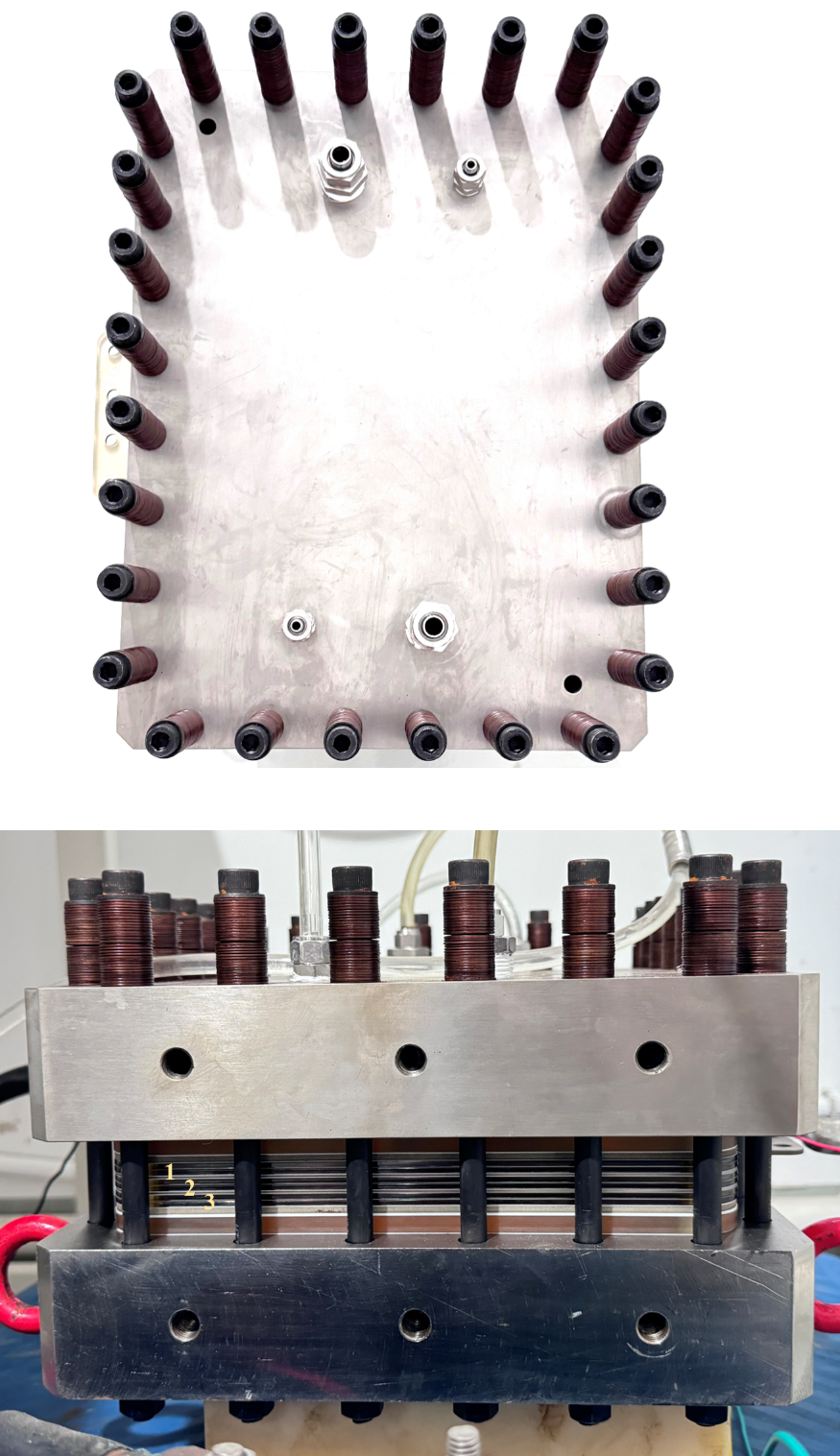
**

**Figure S31.** Photos of the three-cell stack (series connection; 320 cm^2^ per cell).


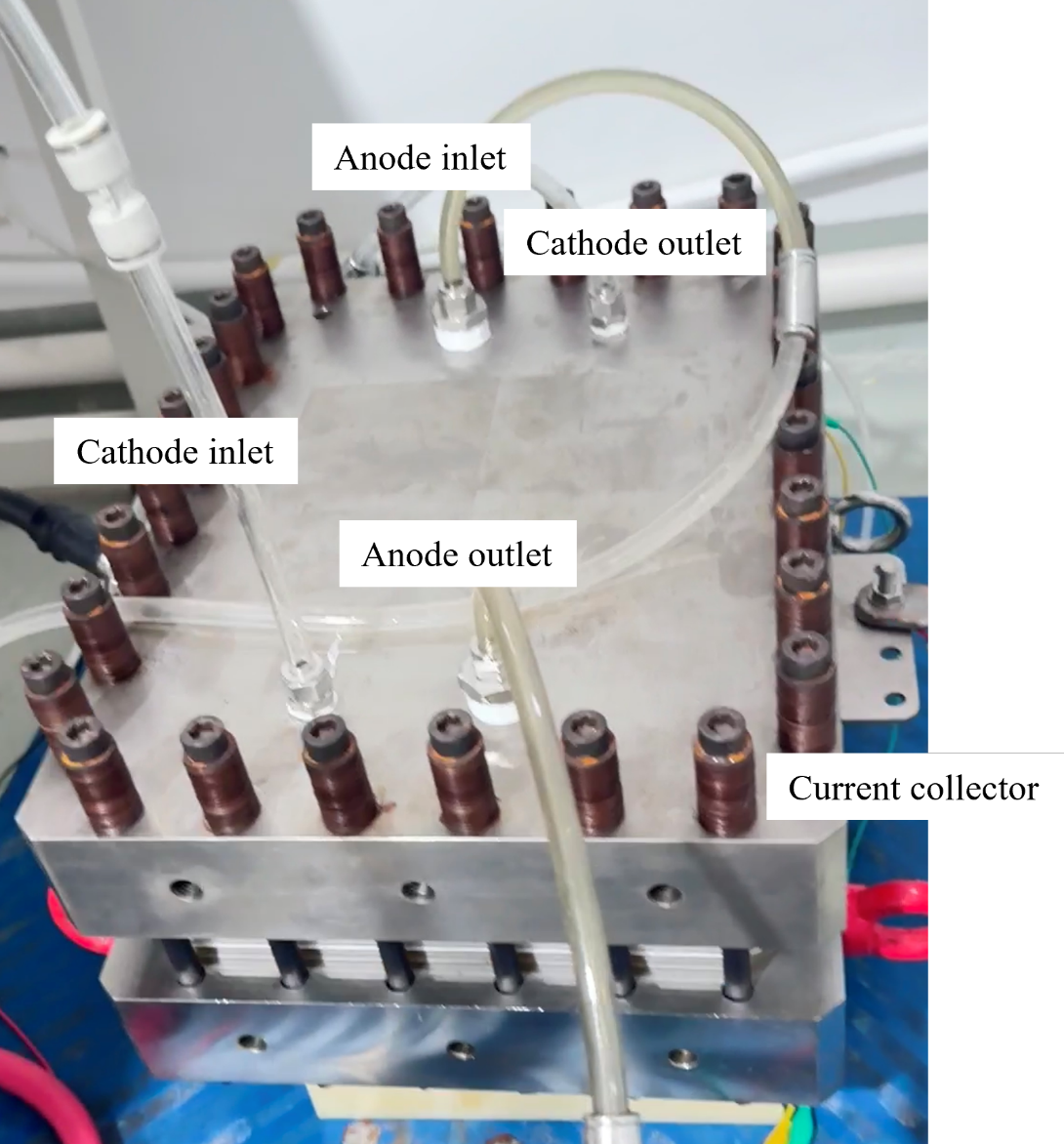


**Figure S32.** Photos of the three-cell stack during testing.


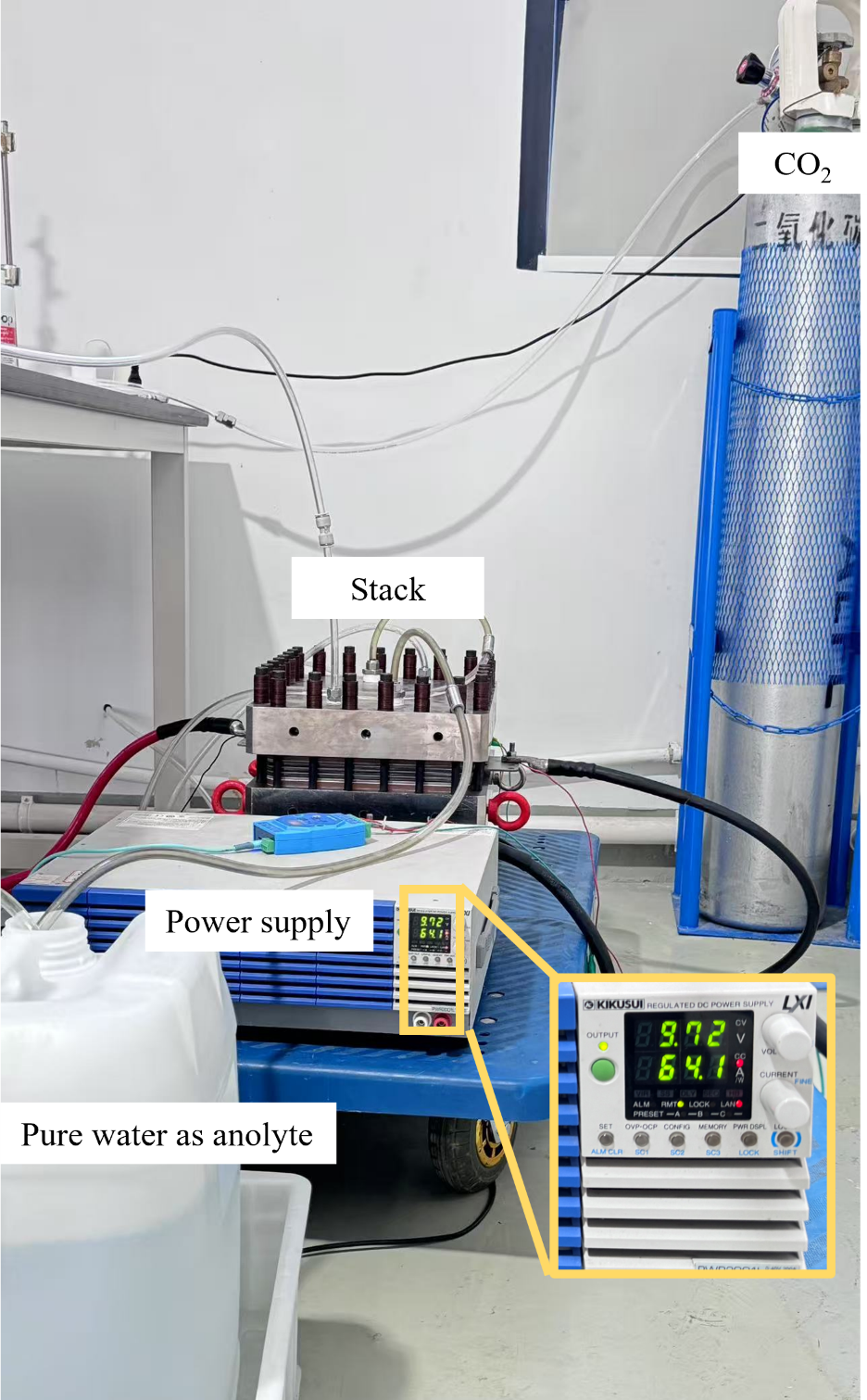


**Figure S33.** Photos of the test setup of three-cell stack.

**Supplementary Table**

**Table S1.** CO_2_RR performance in pure water: comparison of different groups and this work at different operating conditions.

|  | Current density  (mA cm^-2^) | FECO  (%) | Operating temperature  (℃) | Ref. |
| --- | --- | --- | --- | --- |
|  | 20 | 44 | 60 | ^[5]^ |
|  | 40 | 82 |  |  |
|  | 55 | 91 |  |  |
|  | 110 | 94 |  |  |
|  | 200 | 93 |  |  |
|  | 300 | 92 |  |  |
|  | 400 | 91 |  |  |
|  | 500 | 90 |  |  |
|  | 60 | 69 | 25 | ^[6]^ |
|  | 100 | 78 |  |  |
|  | 200 | 55 |  |  |
|  | 260 | 40 |  |  |
|  | 60 | 78 | 40 |  |
|  | 100 | 77.5 |  |  |
|  | 200 | 56 |  |  |
|  | 260 | 45 |  |  |
|  | 60 | 59 | 60 |  |
|  | 100 | 63 |  |  |
|  | 200 | 48 |  |  |
|  | 260 | 38 |  |  |
|  | 50 | 87 | 50 | ^[7]^ |
|  | 100 | 91 |  |  |
|  | 200 | 95 |  |  |
|  | 250 | 95.5 |  |  |
|  | 300 | 95 |  |  |
|  | 350 | 92.6 |  |  |
|  | 400 | 87 |  |  |
|  | 100 | 90 | 50 | ^[8]^ |
|  | 200 | 82 |  |  |
|  | 300 | 81 |  |  |
|  | 100 | 91 | 40 | ^[9]^ |
|  | 200 | 90 |  |  |
|  | 300 | 80 |  |  |
|  | 400 | 75 |  |  |
|  | 500 | 70 |  |  |
|  | 50 | 90 | 40 | ^[10]^ |
|  | 100 | 85 |  |  |
|  | 150 | 38 |  |  |
|  | 100 | 99.9 | 25 | This work |
|  | 200 | 99.9 |  |  |
|  | 300 | 98.9 |  |  |
|  | 400 | 99.4 |  |  |
|  | 500 | 98.9 |  |  |
|  | 100 | 99.0 | 60 |  |
|  | 200 | 98.4 |  |  |
|  | 300 | 97.6 |  |  |
|  | 400 | 96.4 |  |  |
|  | 500 | 95.5 |  |  |
|  | 600 | 92.6 |  |  |
|  | 700 | 87.7 |  |  |
|  | 800 | 76.6 |  |  |
|  | 900 | 80.2 |  |  |
|  | 1000 | 76.4 |  |  |

**Table S2.** Porosity metrics between DFC-I and RTFC-I.

| Metric | DFC-I | RTFC-I | Change |
| --- | --- | --- | --- |
| BET surface area (m^2^ g^-1^) | 4.6730 | 6.8606 | +46.8% |
| Total pore volume, adsorption, pores < 40.3122 nm at P/P_0_ = 0.95 (cm^3^ g^-1^) | 0.008959 | 0.015313 | +70.9% |
| Total pore volume, desorption, pores < 40.3122 nm at P/P_0_ = 0.95 (cm^3^ g^-1^) | 0.010990 | 0.023946 | +117.9% |
| BJH cumulative pore volume (adsorption), 1.7-300 nm (cm^3^ g^-1^) | 0.039243 | 0.049710 | +26.7% |
| BET monolayer capacity Qm (cm^3^ g^-1^ STP) | 1.0736 | 1.5762 | +46.8% |
| BET C constant | 56.356631 | 81.047175 | +43.8% |
| DFT pore volume in pores < 2.002 nm | 0.00013 | 0.00045 | +246% |
| BET fit correlation coefficient | 0.9984605 | 0.9983493 | comparable |

**References**

[1] Q. Wan, L. Yuan, W. Jiang, Y. Liu, L. Zhang, X. Zhuang, J. Zhang, C. Ke, *ACS Sustainable Chemistry & Engineering* **2023**, *11*, 17046-17052.

[2] G. Kresse, J. Furthmüller, *Physical Review B* **1996**, *54*, 11169-11186.

[3] J. P. Perdew, K. Burke, M. Ernzerhof, *Physical Review Letters* **1996**, *77*, 3865-3868.

[4] S. Grimme, J. Antony, S. Ehrlich, H. Krieg, *The Journal of Chemical Physics* **2010**, *132*, 154104.

[5] Z. Yin, H. Peng, X. Wei, H. Zhou, J. Gong, M. Huai, L. Xiao, G. Wang, J. Lu, L. Zhuang, *Energy & Environmental Science* **2019**, *12*, 2455-2462.

[6] J. Fan, B. Pan, J. Wu, C. Shao, Z. Wen, Y. Yan, Y. Wang, Y. Li, *Angewandte Chemie International Edition* **2024**, *63*, e202317828.

[7] J. Chen, W. Niu, L. Xue, K. Sun, X. Yang, X. Zhang, W. Li, S. Huang, W. Shi, B. Zhang, *Nanoscale* **2024**, *16*, 16510-16516.

[8] R. Xue, S. Yuan, R. Wang, T. Bi, G. Zhang, H. Li, J. Yin, L. Luo, S. Shen, X. Yan, J. Zhang, *Journal of Energy Chemistry* **2025**, *108*, 390-399.

[9] M. Heßelmann, J. K. Lee, S. Chae, A. Tricker, R. G. Keller, M. Wessling, J. Su, D. Kushner, A. Z. Weber, X. Peng, *ACS Applied Materials & Interfaces* **2024**, *16*, 24649-24659.

[10] X. Huang, L. Qian, Y. Ji, W. Niu, Y. Hu, L. Xue, J. Li, S. Huang, J. Zhang, Y. Li, B. Zhang, *Chem Catalysis* **2022***,* *6*, 92-133.
